# Selective Ablation of the Androgen Receptor in Mouse Sertoli Cells Affects Sertoli Cell Maturation, Barrier Formation and Cytoskeletal Development

**DOI:** 10.1371/journal.pone.0014168

**Published:** 2010-11-30

**Authors:** Ariane Willems, Sergio R. Batlouni, Arantza Esnal, Johannes V. Swinnen, Philippa T. K. Saunders, Richard M. Sharpe, Luiz R. França, Karel De Gendt, Guido Verhoeven

**Affiliations:** 1 Laboratory for Experimental Medicine and Endocrinology, Katholieke Universiteit Leuven, Leuven, Belgium; 2 Aquaculture Center (CAUNESP), São Paulo State University, Jaboticabal, São Paulo, Brazil; 3 Medical Research Council Human Reproductive Sciences Unit, Centre for Reproductive Biology, Edinburgh, Scotland, United Kingdom; 4 Laboratory of Cellular Biology, Department of Morphology, Institute of Biological Sciences, Federal University of Minas Gerais, Belo Horizonte, Minas Gerais, Brazil; Temasek Life Sciences Laboratory, Singapore

## Abstract

The observation that mice with a selective ablation of the androgen receptor (AR) in Sertoli cells (SC) (SCARKO mice) display a complete block in meiosis supports the contention that SC play a pivotal role in the control of germ cell development by androgens. To delineate the physiological and molecular mechanism responsible for this control, we compared tubular development in pubertal SCARKO mice and littermate controls. Particular attention was paid to differences in SC maturation, SC barrier formation and cytoskeletal organization and to the molecular mediators potentially involved. Functional analysis of SC barrier development by hypertonic perfusion and lanthanum permeation techniques and immunohistochemical analysis of junction formation showed that SCARKO mice still attempt to produce a barrier separating basal and adluminal compartment but that barrier formation is delayed and defective. Defective barrier formation was accompanied by disturbances in SC nuclear maturation (immature shape, absence of prominent, tripartite nucleoli) and SC polarization (aberrant positioning of SC nuclei and cytoskeletal elements such as vimentin). Quantitative RT-PCR was used to study the transcript levels of genes potentially related to the described phenomena between day 8 and 35. Differences in the expression of SC genes known to play a role in junction formation could be shown from day 8 for *Cldn11*, from day 15 for *Cldn3* and *Espn*, from day 20 for *Cdh2* and *Jam3* and from day 35 for *ZO-1*. Marked differences were also noted in the transcript levels of several genes that are also related to cell adhesion and cytoskeletal dynamics but that have not yet been studied in SC (*Actn3, Ank3, Anxa9, Scin, Emb, Mpzl2*). It is concluded that absence of a functional AR in SC impedes the remodeling of testicular tubules expected at the onset of spermatogenesis and interferes with the creation of the specific environment needed for germ cell development.

## Introduction

Androgens play a pivotal role in the control of spermatogenesis. Under a number of conditions they are even able to maintain fertility in the virtual absence of follicle stimulating hormone (FSH) [1]–[5]. Interestingly, most data indicate that the androgen receptor (AR) is not expressed in germ cells (GC) and that GC can develop normally in the absence of cell-autonomous AR expression [6]–[9]. This indicates that androgens affect GC development indirectly by acting on somatic testicular cells. The development of animals with cell-selective knockouts of the AR in various somatic testicular cells points to the Sertoli cell (SC) as the key mediator of the effects of androgens in the control of spermatogenesis. In fact, mice with a selective knockout of the AR in SC (SCARKO) develop a complete block in meiosis [10], [11]. Data on the consequences of a knockout of the AR in peritubular myoid cells (PTM) are more controversial. Whereas original studies suggested that the latter knockout has only minor effects on GC development [12], more recent data indicate that those studies mainly targeted vascular smooth muscle cells and that more appropriate targeting of the AR in PTM (using a line of mice expressing Cre more specifically in PTM) causes major disturbances in spermatogenesis [13]. Notably, secondary disturbances in SC and Leydig cells (LC) function appear to be one consequence of the ablation of the AR in PTM highlighting again the carefully balanced dialogue that exists between the different somatic cell types within the testis and the pivotal role the SC plays in orchestrating full, functional maturation of the GC cohort.

Although our studies as well as those of others [1], [10], [11], [14] agree that AR-dependent modulation of SC function plays a critical regulatory role in supporting GC as they mature within the seminiferous epithelium, we are still some way from fully understanding the molecular mediators and pathways by which androgens orchestrate the relationship between SC and GC. Unfortunately, studies on isolated and cultured SC or SC-lines have proven of limited use since these cells appear to de-differentiate in the absence of GC, exhibit a marked reduction in androgen responsiveness and lack the expression of prototypic androgen-regulated genes such as reproductive homeobox 5 (*Rhox5*) [15], [16]. Various attempts have been made to identify androgen-regulated genes under *in vivo* conditions that maintain the normal cellular microenvironment of the SC. Examples include: microarray analysis of the impact of exogenously administered androgens on gene expression in the testis of prepubertal mice or mice that are hypogonadal due to a large deletion of the gonadotropin-releasing hormone 1 gene (*Gnrh1; hpg* mice) [17], [18] and identification of testicular genes that are differentially expressed (and putatively androgen regulated) in SCARKO mice or mice with a ubiquitous inactivation of the AR (testicular feminization mutation; *Tfm*) versus wild-type controls [19]–[21]. Although there was very limited overlap between the series of androgen-regulated genes identified in these studies, genes related to particular processes and functions (including: protease balance, cell interactions, cytoskeletal dynamics) were prominently present [19]–[22].

Whereas in other testicular somatic cells (PTM, interstitial cells) AR expression is already established during embryonic life, AR expression in SC starts quite late (postnatal day 4–5 in rats and mice, 4–8 years of age in boys) [23]–[25]. Recent data indicate that very soon after the appearance of the AR selected genes become activated in mouse SC [23] and that on day 10 the expression of as many as 692 genes is affected by the presence of an active AR in SC [19]. Functional analysis revealed a prominent presence of genes encoding proteases and protease inhibitors, cell adhesion molecules, extracellular matrix elements and cytoskeletal molecules potentially related to tubular restructuring and changes in cell junction dynamics. Based upon this observation the hypothesis was advanced that, during early puberty, androgens may contribute to the creation of a specific environment needed for GC development [19]. To test this hypothesis we compared tubular development, SC maturation, cytoskeletal changes and expression of genes potentially related to these events in wild type and SCARKO mice. Particular attention was devoted to the development of the SC (blood-testis) barrier, a structure that ultimately divides the tubules into a basal compartment (containing mainly spermatogonia) and an adluminal compartment (providing the specific microenvironment needed for meiotic and postmeiotic stages of germ cell development) but that also permits controlled passage of preleptotene spermatocytes from the basal to the adluminal compartment. The mature SC barrier is a complex, well organized and dynamic structure containing tight junction proteins (e.g. occludins, claudins, junction adhesion molecules), anchoring junction proteins (e.g. N-cadherin, espin) and gap junction proteins (e.g. connexin 43). A network of adaptor proteins (e.g. zonula occludens protein 1, -2 and 3) links these membranous components to intermediate filaments and to the filamentous actin (F-actin) cytoskeleton [26], [27]. A role for AR regulated gene expression in the formation of the SC barrier has been inferred from reports indicating that it is functionally defective in *Tfm* mice [28] and also in Ar^flox(ex1-neo)/Y^; AMH-Cre mice, a mouse model with an ablation of the AR in SC as well as a marked reduction in AR expression in all other AR expressing cells [29], [30]. However in SCARKO mice some tubules within the adult testis contain an identifiable lumen or small amounts of fluid accumulation, calling into question the requirement of the AR in SC for the formation of a functional SC barrier [10].

A detailed analysis of tubular development in SCARKO and control mice shows that ablation of the AR in SC results in delayed and defective formation of the SC barrier. This defect is accompanied by defective SC maturation including signs of nuclear immaturity, a failure of the nuclei to descend to the base of the tubules and disturbed development of the cytoskeleton. The observed morphological defects are accompanied by disturbances in the expression and localization of previously identified and novel molecules related to cell adhesion/interaction and cytoskeletal dynamics.

## Materials and Methods

### Ethics statement

All animals were treated according to the National Institutes of Health Guide for the Care and Use of Laboratory Animals, and all experiments were approved by the “Ethical Committee Animal Tests” of the Catholic University of Leuven (project licence number 004/2006).

### Generation of transgenic mice

Mice with a Sertoli cell-selective knockout of the AR (SCARKO) were generated by crossing female mice (98% CD1) heterozygous for a floxed AR allele (*AR^flox/+^*) with male mice (C57BL/6SJL) carrying a Cyclization Recombination (Cre) recombinase of which the expression is controlled by the SC-specific anti-Müllerian hormone promotor (AMH-Cre) transgene [31], kindly provided by Dr. F. Guillou (Tours, France). Littermate males carrying only the AMH-Cre transgene were used as a control for the SCARKO animals. The genotype of control and SCARKO animals was confirmed by PCR on tailtips as described elsewhere [10].

### Hypertonic perfusions

Hypertonic perfusions of testes were performed essentially as described [32]. Peripubertal (10-, 15-, 25- and 35-day-old) and adult (≥50-day-old) mice (at least 3 SCARKOs and 3 controls at each age) were anaesthetized with a ketamin-xylazine-heparin solution (100 mg/kg body weight, 15 mg/kg body weight and 1000 IU/kg body weight, respectively) injected intraperitoneally 20 min prior to perfusion. Testes were perfused by intracardiac injection of 10% glucose in 0.9% NaCl for 10 min followed by 30 min perfusion with fixative containing 10% glucose and 3% glutaraldehyde buffered in 0.05 M sodium cacodylate (pH 7.4). After perfusion, testes were removed and postfixed in the above mentioned fixative. After 24 h, testes were washed in 70% ethanol, followed by several washes in 100% ethanol. After dehydration, testes were embedded in methyl methacrylate (MMA), 2 µm sections were cut with a Leica RM 2155 microtome with a tungsten carbide D-profile microtome knife and stained with toluidine blue.

### Immersion fixation with lanthanum and electron microscopic evaluation

Fixative containing lanthanum (pH 7.3) was prepared essentially as described [33]. Lanthanum hydroxide (2%; pH 7.8 with 0.01 N NaOH) was gently mixed with an equal volume of 4% glutaraldehyde in 0.2 M sodium cacodylate (pH 7.3 with 1N HCl). Mice were killed by cervical dislocation, testes were removed and decapsulated and pre-fixed for 5 min in fixative containing lanthanum. After pre-fixation, testes were cut into small pieces (2–3 mm^3^) and immersed in the fixative containing lanthanum for 5 h at room temperature. Afterwards, samples were rinsed and stored in buffered lanthanum solution (1% lanthanum in 0.1 M sodium cacodylate, pH 7.3) at 4°C until further processing.

For transmission electron microscopy analysis testis samples were post-fixed in 1% osmium tetroxide in sodium cacodylate, pH 7.2 for 2 h at 4°C and immersed in 2% uranyl acetate solution at 4°C overnight. The samples were dehydrated in ethanol and embedded in Epon 812-Araldite 502. Thin sections were then routinely prepared for light microscopy analysis. Ultra-thin sections (70–80 nm) from the previously selected areas of the testes were mounted on 200-mesh uncoated copper grids, stained with 2% uranyl acetate in distilled water for 1 h and 0.5% lead citrate in distilled water for 30 min. These grids were examined in a 100 CX-II Jeol transmission electron microscope (80 kV).

### Immunohistochemistry

Testes derived from 20-, 25-, 35- and 50-day-old SCARKO and control mice were either fixed in Bouin's fluid for 6 hours and stored in 70% ethanol at 4°C or frozen in isopentane and liquid nitrogen and stored at −80°C until further processing.

Fluorescent colocalization of zonula occludens 1 (ZO-1) and F-actin were carried out on sections of frozen testes. Frozen testes were embedded in Neg-50 Frozen Section Medium (Thermo Fisher Scientific), cut at 10 µm with a cryostat and mounted on Superfrost® Plus slides (Thermo Scientific). Sides were fixed for 10 min at room temperature (RT) with 4% paraformaldehyde and washed 2 times with 1×Dulbecco's Phosphate Buffered Saline (DPBS; Invitrogen). Afterwards, sections were permeabilized with 0.5% Triton X-100 for 15 min and washed 3 times with DPBS. Next, 4 drops of Image-iT™ Fx Signal Enhancer (Invitrogen) were applied upon tissue sections for 30 min (at RT) and again washed 3 times with 1×DPBS. Subsequently sections were blocked (30 min; RT) with 1×DPBS supplemented with 10% normal goat serum and 1% BSA, washed 1 time with 1×DPBS followed by overnight incubation with rabbit anti-ZO-1 antibody (1/500; 40-2200 from Invitrogen) in blocking buffer. Before and after incubation with secondary antibody (Alexa fluor® 488 goat anti-rabbit IgG (H+L) at 2 µg/ml; A-11008 from Invitrogen) and phalloidin-TRITC (P1951 from SIGMA at 0.2 µg/ml) for 1 hour at RT in blocking buffer, slides were washed 3 times in 1×DPBS. Next, slides were incubated for 10 min with 4′,6′-diamino-2-phenylindole (DAPI) (5 µg/ml; D1306 from Invitrogen) in 1×DPBS, once more washed 3 times with 1×DPBS and mounted in Fluorescent Mounting Medium (S3023, DakoCytomation). Fluorescent images were captured using a Leica DMR microscope with a Sony DXC-9100P camera.

Fluorescent colocalization of vimentin (VIM) and GATA binding protein 1 (GATA1) or connexin 43 (CX43) and espin (ESPN) were carried out on fixed tissue sections using standard protocols [34], [35]. All washes were carried out using phosphate buffered saline and negative controls were performed using serum from the same species in which primary antibodies were raised. Briefly, sections were incubated overnight at 4°C with anti-VIM (1/100; ab7783 from Abcam) or anti-CX43 (1/100; 71-0700 from Invitrogen), washed and incubated with the appropriate secondary antibodies (Alexa fluor® 488 goat anti-rabbit at 1/200 from Molecular Probes and biotinylated goat anti-rabbit at 1/500 from Dako respectively). Sections incubated with anti-CX43 were also incubated with strepavidin-conjugated alexa 546 (Molecular Probes, Poortgebouw, Holland). All non-specific binding sites were blocked with appropriate normal serum/PBS/BSA for 30 min, then sections were again incubated overnight this time with anti-GATA1 (1/30; sc266 from Santa Cruz Biotechnology) or anti-ESPN (1/30; 611656 from BD Bioscience) respectively. After further washes these antibodies were detected with suitable secondary antibodies (rabbit anti-rat peroxidase at 1/200 from Sigma and Alexa fluor® 488 goat anti-mouse at 1/200 from Molecular Probes respectively). Sections incubated with anti-GATA1 were also incubated with tyramide Cy3 (TSA plus cyanine 3 system from Perkin-Elmer Life Sciences, Boston, MA). Sections were counterstained by incubating them for 10 min with DAPI (Sigma) diluted 1/1000 in PBS and mounted in Permafluor (Beckman Coulter, High Wycombe, UK) aqueous mounting medium.

Tissue sections from 3 animals in each group were run simultaneously. To ensure direct comparability of staining intensities, one section each from control and SCARKO mice were mounted on the same slide.

### RNA Extraction and quantitative RT-PCR

RNA was prepared from testes derived form SCARKO and control animals at the indicated ages. Mice were killed by cervical dislocation, testes were snap-frozen in liquid nitrogen immediately after removal and stored at −80°C. Before RNA extraction, testes were weighed and homogenized in a Dounce homogenizer (Kontes Co., Vineland, NJ). RNA was isolated with the RNeasy® Mini kit (Qiagen, Chatsworth, CA) according to the manufacturer's instructions, encompassing an on-column deoxyribonuclease I (DNase I) treatment of the RNA. Five ng of *luciferase* mRNA (Promega, Madison, WI) was added to the whole testis sample at the start of the RNA extraction procedure to control for the efficiency of RNA extraction, RNA degradation and the reverse transcription step and to allow specific mRNA levels to be expressed per testis [36].

cDNA was synthesized from 1 µg RNA using Superscript II RT, RNaseOUT®TM and random hexamer primers (Invitrogen Life Technologies, Inc) according to the manufacturer's protocol. For quantification of gene expression, the 7500 Fast Real-Time PCR system (Applied Biosystems, Foster City, CA) was used running the ‘Fast RT-PCR’ protocol (2 min at 50°C, 2 min at 95°C and 40 cycles of 3 sec at 95°C and 30 sec at 60°C). Quantitative real-time PCR (qPCR) components for *synaptonemal complex protein 3* (*Sycp3*), *steroid 17-α- hydroxylase/17,20-lyase* (*Cyp17a1*), *claudin 11* (*Cldn11*), *claudin 3* (*Cldn3*), *ZO-1*, *luciferase* and *18S ribosomal RNA* (*Rn18S*) were obtained from the Platinum® SYBR® Green qPCR SuperMix-UDG kit (Invitrogen). For qPCR assays with fluorescent probe detection (*Rhox5*, *actinin α3* (*Actn3*), *ankyrin3* (*Ank3*), *annexin A9* (*Anxa9*), *scinderin* (*Scin*), *embigin* (*Emb*), *myelin protein zero-like2* (*Mpzl2*), *occludin* (*Ocln*), *junction adhesion molecule 3* (*Jam3*), *Vim*, *Cx43*, *Espn* and *N-cadherin* (*Cdh2*)) the TaqMan® Fast Universal PCR Master Mix (2x) (Applied Biosystems) was used as described elsewhere [23]. Sequences of primers, probes and identification numbers of TaqMan® Gene Expression Assays (Applied Biosystems) are described in Table 1 and Table 2.

**Table 1:** Oligonucleotide primers and probes used for qPCR.

Gene name	Gene	Accession	5′ Primer (Fw)
	symbol	number	3′ Primer (Rv)
			Probe
Reproductive	*Rhox5*	NM_008818	Fw: 5′-TCATCATTGATCCTATTCAGGGTATG-3′
homeobox 5			Rv: 5′-CTCTCCAGCCTGGAAGAAAGC-3′
			Probe: 5′-6-FAM-CTCGGAAGAACAGCATGATGTGAAAGCA-TAMRA-3′
Synaptonemal	*Sycp3*	NM_011517	Fw: 5′-ATGCTTCGAGGGTGTGGG-3′
complex protein 3			Rv: 5′-TTCCACCAGGCACCATCTTT-3′
Steroid 17-α-	*Cyp17a1*	NM_007809	Fw: 5′-GGGCACTGCATCACGATAAA-3′
hydroxylase/17,20-lyase			Rv: 5′-GATCTAAGAAGCGCTCAGGCA-3′
Claudin 11	*Cldn11*	NM_008770	Fw: 5′-CGTCATGGCCACTGGTCTCT-3′
			Rv: 5′-GGCTCTACAAGCCTGCACGTA-3′
Claudin 3	*Cldn3*	NM_009902	Fw: 5′-GCGCCTTGCTGTGTTGCT-3′
			Rv: 5′-AGAGGATCTTGGTGGGTGCAT-3′
Zonula occludens 1	*ZO-1*	NM_001163574	Fw: 5′-GGAGCTACGCTTGCCACACT-3′
			Rv: 5′-GGTCAATCAGGACAGAAACACAGT-3′
*Luciferase*	*Luciferase*	L4561(from	Fw: 5′-TCGAAGTATTCCGCGTACGTG-3′
		Promega)	Rv: 5′-GCCCTGGTTCCTGGAACAA-3′
18S ribosomal RNA	*Rn18S*	NR_003278	Fw: 5′-CGCCGCTAGAGGTGAAATTC-3′
			Rv: 5′-TTGGCAAATGCTTTCGCTC-3′

**Table 2:** TaqMan® Gene Expression Assays used for qPCR.

Gene name	Gene	Accession	TaqMan® Gene Expression
	symbol	number	Assay (Applied Biosystems)
Actinin, alfa 3	*Actn3*	NM_013456	Mm00496495_m1
Ankyrin 3, node of	*Ank3*	NM_009670	Mm00464776_m1
Ranvier (ankyrin G)			
Annexin A9	*Anxa9*	NM_023628	Mm00499250_m1
Scinderin	*Scin*	NM_009132	Mm00485972_m1
Embigin	*Emb*	NM_010330	Mm00515881_m1
Myelin protein zero-like 2	*Mpzl2*	NM_007962	Mm00468397_m1
Occludin	*Ocln*	NM_008756	Mm00500912_m1
Junction adhesion	*Jam3*	NM_023277	Mm00499214_m1
molecule 3			
Vimentin	*Vim*	NM_011701	Mm00449208_m1
Connexin 43 (gap junction	*Cx43 (Gja1)*	NM_010288	Mm00439105_m1
protein, alpha 1, 43kDa)			
Espin	*Espn*	NM_019585	Mm02026930_s1
N-cadherin	*Cdh2*	NM_007664	Mm00483213_m1

The quantity of target mRNA of cell adhesion molecules and cytoskeletal elements in whole testis extracts of SCARKO and control mice was normalized to an external *luciferase* mRNA standard, added before RNA extraction (as described above). For localization of transcripts in different cell fractions, the quantity of target mRNA was normalized to *Rn18S*. Results were quantified by the comparative threshold method [37] and ΔΔC_t_ values were expressed relative to the average value of the control on day 10 (arbitrarily given a value of 100) for the evaluation of expression levels from day 8 to day 35. For the experiments in which the SC where enriched, ΔΔC_t_ values were expressed relative to the average value of whole testis extract (arbitrarily given a value of 100). All samples were run in triplicate. Microarray data reflecting the expression pattern of cell adhesion molecules and cytoskeletal elements (day 8–day 20) were obtained as described previously [19]. Raw data of this microarray study are available at the GEO website (GSE2259).

### Statistical analysis

qPCR time-studies were analysed by a two-way ANOVA supplemented with a Fisher multiple comparison test using NCSS2000 software (NCSS Statistical Analysis and Data Analysis Software, Kaysville, UT, USA). A p-value ≤ 0.05 was considered statistically significant.

## Results

### Evidence for marked differences in barrier function in SCARKO and control testes

We have previously reported that the reduction in testis size in SCARKO mice as compared to littermate controls is accompanied by a striking decrease in tubular diameter (down to 61% of the control on day 50) and an even more marked decrease in lumen formation (down to 32% of the control when expressed as a percentage of testis volume) [10]. Here we used efferent duct ligation to explore the ability of SCARKO testes to secrete and accumulate testicular fluid. In control mice, unilateral ligation of the efferent ducts for 24 h resulted in a 19% increase in weight of the ligated testis as compared to the unligated testis (*p*<0.05) reflecting active fluid secretion and an intact SC barrier. However in SCARKO mice, a 7% decrease in testis weight (not significant) rather than an increase was observed, suggesting that SC fluid secretion and/or barrier formation were impaired (Table S1).

**Figure 1:**
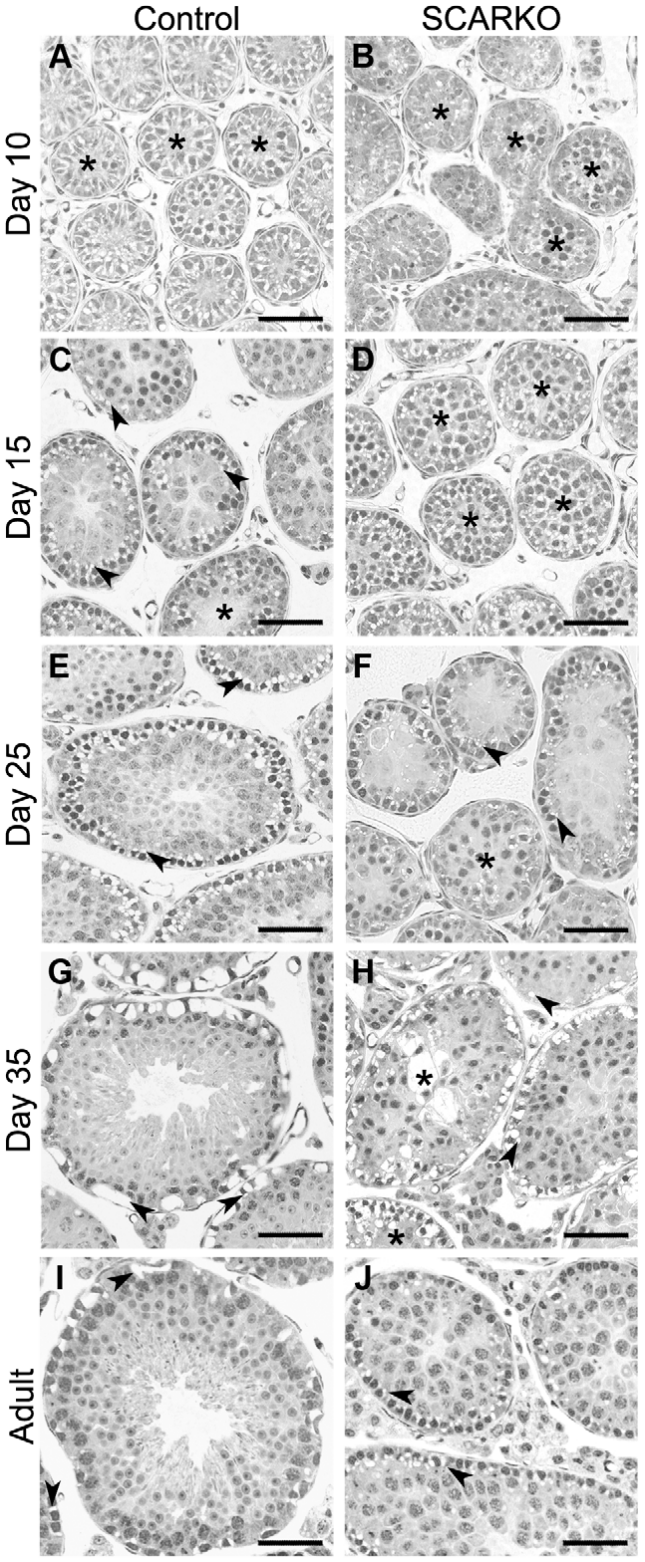
Use of hypertonic perfusion to assess the integrity of the SC barrier. Testes from mice with a SC-selective knockout of the androgen receptor (SCARKO; panel B, D, F, H, J) and from control littermates (panel A, C, E, G, I) were subjected to hypertonic perfusion at day 10 (panel A, B), day 15 (panel C, D), day 25 (panel E, F), day 35 (panel G, H) and at adult age (panel I, J) as described in the Methods section. At least 3 animals were studied at each age and representative pictures have been selected. Black asterisks indicate representative tubules with shrinkage around cells that are centrally positioned (no functional barrier). Arrowheads indicate basal compartment shrinkage (presence of a functional barrier). Scale bars = 50 µm.

Perfusion with hypertonic fluid [32] was used to evaluate more specifically the formation of a functional SC barrier in testes of peripubertal (day 10, 15, 25, and 35) and adult SCARKO and control littermates (Figure 1). At least 3 animals were studied at each of these time points. Despite some variability between individual animals, as also reported by other investigators [32], a consistent evolution of barrier formation was observed. At the age of 10 days all tubular cells, in control (Figure 1A) as well as in SCARKO mice (Figure 1B), displayed hypertonicity-related shrinkage, indicating the absence of a functional barrier. On day 15, barrier formation had occurred in most of the tubules from control mice as evidenced by limitation of cell shrinkage to the basal compartment (Figure 1C). In SCARKO mice of the same age no barrier formation was observed (Figure 1D). In 25-day-old control animals barrier formation was noted in all testicular tubules (Figure 1E). In SCARKO mice of the same age the situation was more variable with barrier formation in some tubules (particularly in those displaying pachytene spermatocyte development) and absence of barrier formation in others (Figure 1F). A similar picture was noted on day 35 (Figure 1G, H). In some tubules of SCARKO mice, hypertonicity-induced shrinkage of centrally located cells was seen despite clear signs of (partial) barrier formation at the base of the tubules (Figure 1H). At adult age, barrier formation was observed in all tubules of control mice (Figure 1I) and in nearly all tubules of SCARKO mice (Figure 1J). Quantification of the fraction of tubules with a fully functional barrier (Figure S1) confirmed the differences in development between SCARKO and control testes but also showed that in SCARKO mice the fraction of tubules with an intact barrier increased from 18.2% at day 25 to 60.1% at day 35 and to 84.7% at adult age. In adult control mice 99.7% of the tubules displayed an intact barrier.

### Electron microscopic demonstration of delayed and incomplete barrier formation in SCARKO testes

**Figure 2:**
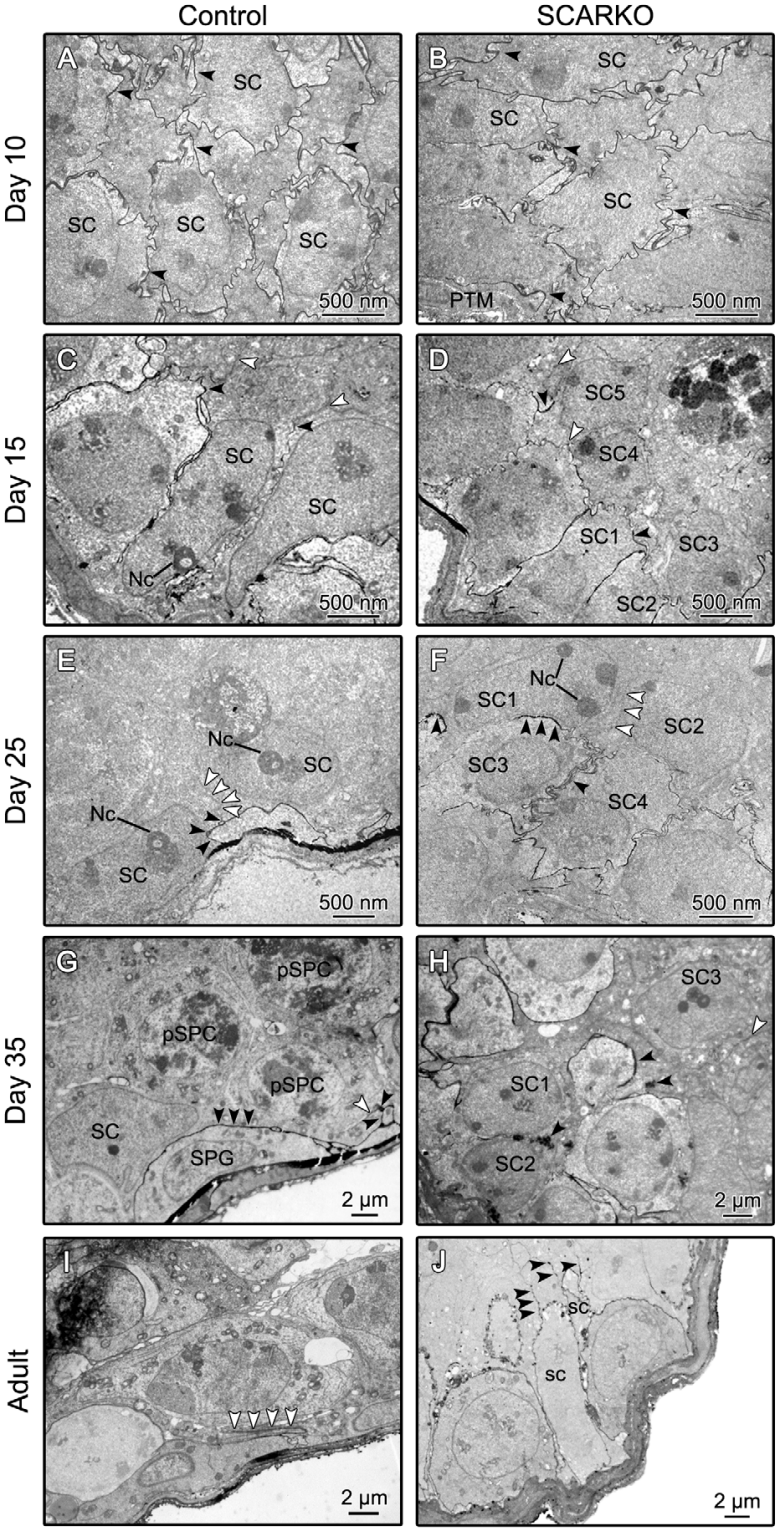
Comparison of barrier formation in 10-, 15-, 25-, 35-day-old and adult control and SCARKO testes. Immersion fixation with lanthanum and electron microscopic evaluation of testes from mice with a SC-selective knockout of the androgen receptor (SCARKO; panel B, D, F, H and J) and from control littermates (panel A, C, E, G and I) were performed as described in the Methods section. At least 3 animals were studied at each age and representative pictures have been selected. In testes derived from 10-day-old mice lanthanum (black arrowheads) is seen in the intercellular spaces throughout all the seminiferous tubules reflecting absence of barrier formation in control as well as SCARKO testes (panel A and B respectively). Initial formation of tight junctions and a Sertoli cell (SC) barrier can be seen in 15-day-old control and SCARKO testes (panel C and D). In control testes mature Sertoli cells (SC) are lining the tubular lamina. Tight junction and barrier formation (white arrowheads) inhibits penetration of lanthanum (black arrowheads) into the adluminal compartment. In SCARKO tubules peripherally located SC (panel D: SC1, SC2, SC3), displaying signs of immaturity, allow penetration of lanthanum (black arrowheads). Formation of a tight junction barrier may be noted between more centrally located SC (panel D: SC4, SC5) that also display more advanced signs of maturation. At the age of 25 days barrier formation (white arrowheads) is evident in all tubules from control animals and in some tubules from SCARKO mice. In control testes mature SC lining the basal lamina produce a lanthanum impermeable barrier (panel E). In some SCARKO tubules tight junction and barrier formation is observed between more centrally located SC (panel F: SC1, SC2) showing signs of maturity. Lanthanum permeation is observed between peripherally located less differentiated SC (panel F: SC3, SC4) resulting in a broadened basal compartment. At the age of 35 days restriction of lanthanum to the basal compartment is observed in all tubules of control mice (panel G) and in some tubules of SCARKO mice (panel H). Panel G shows a well developed SC barrier typical for tubules of 35-day-old control mice. Note the presence of primary spermatocytes (pSPC) in the adluminal compartment and a spermatogonium (SPG) close to the basement membrane. In 35-day-old SCARKO tubules permeable tight junctions may be noted between and above immature basally positioned SC (panel H: black arrowhead between SC1 and SC2). Impermeable junctions (white arrowheads) are noticed in the upper region containing more mature SC (panel H: SC3). In adult control mice no permeable tubules were found (panel I). The SC barrier between mature SC (white arrowheads) is usually located in the more basal part of the SC. In adult SCARKO mice however lanthanum could still be observed between and above immature SC (panel J: black arrowheads). Nc: prominent nucleoli; PTM: peritubular myoid cell.

In confirmation of the findings described above, examination of the permeation of lanthanum in 10-day-old control (Figure 2A) and SCARKO (Figure 2B) testes revealed free permeation of the spaces between SC and between SC and GC, indicating absence of barrier formation. On day 15, formation of tight junctions blocking the penetration of lanthanum in the adluminal compartment was observed in the majority of tubules of testes derived from control mice (Figure 2C; white arrowheads) and in some tubules derived from SCARKO mice (Figure 2D; white arrowheads). Interestingly, the SC lining the basal lamina of tubules in control testes appeared more mature (elongated shape, elongated and irregularly shaped nuclei with a prominent and tripartite nucleolus) than those lining the basal lamina in tubules of SCARKO testes. Moreover, in SCARKO tubules tight junctions were observed mainly between more centrally located SC, which also appeared more mature than their peripherally located counterparts. In tubules of 25-day-old control mice only very few SC displayed signs of immaturity and tight junctions restricting permeation of lanthanum were seen between SC located at the base of the epithelium (Figure 2E). Barrier formation could also be observed in some tubules of SCARKO mice (Figure 2F). In these mice, however, tight junctions were frequently dislocated to the more centrally located regions of the seminiferous epithelium that also contained the more mature SC, resulting in a broadening of the basal compartment, compared to that of control mice. Moreover, in SCARKO testes, unlike in control testes, some tubules remained permeable to lanthanum. In 35-day-old control mice a well developed SC barrier preventing passage of lanthanum was seen in all tubules analyzed (Figure 2G). In 35-day-old SCARKO mice the formation of tight junctions and a SC barrier was more prominent than that at 25 days of age but nonetheless in some tubules variable amounts of lanthanum were evident in the adluminal compartment (Figure 2H). In adult control mice passage of lanthanum was blocked in all tubules (Figure 2I) while in adult SCARKO mice the picture resembled that observed in 35-day-old SCARKO testes with variable penetration of lanthanum (Figure 2J).

**Figure 3:**
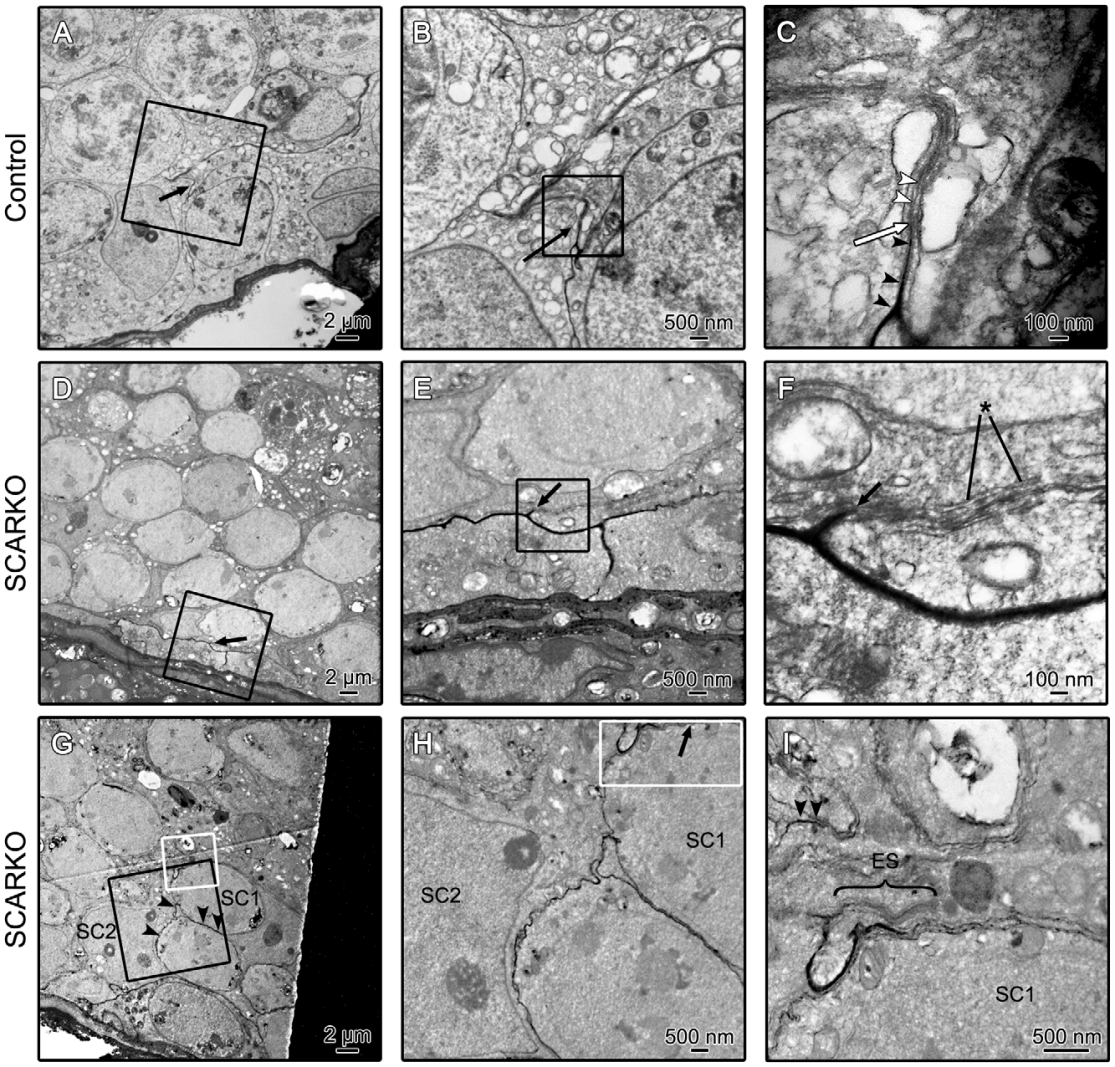
Lanthanum penetration and barrier formation in testes derived from 35-day-old control and SCARKO testes. A typical tight junction impeding further penetration of lanthanum in a day 35 control testis is shown in panel A, B and C. Panel B represents an enlargement of the boxed area in panel A showing an impermeable barrier. A further enlargement of the boxed area in panel B is shown in panel C. Black arrowheads show lanthanum below the tight junction that starts at the white arrow. So called “kissing points” of the tight junction can be seen (white arrowheads) within the ectoplasmic specialization. An impermeable barrier in a day 35 SCARKO testis is illustrated in panel D, E and F (black arrow). Panel E and F represent enlargements of the boxed area's indicated in panel D and E respectively. Cisternae of smooth endoplasmic reticulum (asterisk) surrounding a tight junction may be noted in panel F. Panel G, H and I show another area of the same SCARKO testis where lanthanum penetrates between two SC (SC1 and SC2). Panel H represents an enlargement of the area in the black square in panel G. An enlargement of the more adluminally located area in the white square of panel G is shown in panel I. The bracket in panel I shows an ectoplasmic specialization (ES) that acts as a barrier for further penetration of lanthanum. Nonetheless lanthanum may be noted above this barrier (black arrowheads). Pictures are representative for at least 3 animals studied.

A more detailed investigation of junction formation was performed in control and SCARKO testes of 25-day-old, 35-day-old and adult mice. Representative data from day 35 are summarized in Figure 3. Similar results were observed at the other ages. The data indicate the presence of typical tight junctions blocking the passage of lanthanum in control animals (Figure 3A, B and C). In SCARKO mice formation of impermeable tight junctions was observed in some areas (Figure 3D, E and F) but diverse areas of the epithelium remained permeable to lanthanum despite the presence of similar junctions (Figure 3G, H and I) suggesting that the zonulae occludens that encircle the whole epithelium may contain permeable areas. Further investigations (including freeze fracture studies) will be required to clarify the nature of these defects.

### Immunohistochemical evidence for delayed and incomplete formation of the SC barrier in SCARKO mice

**Figure 4:**
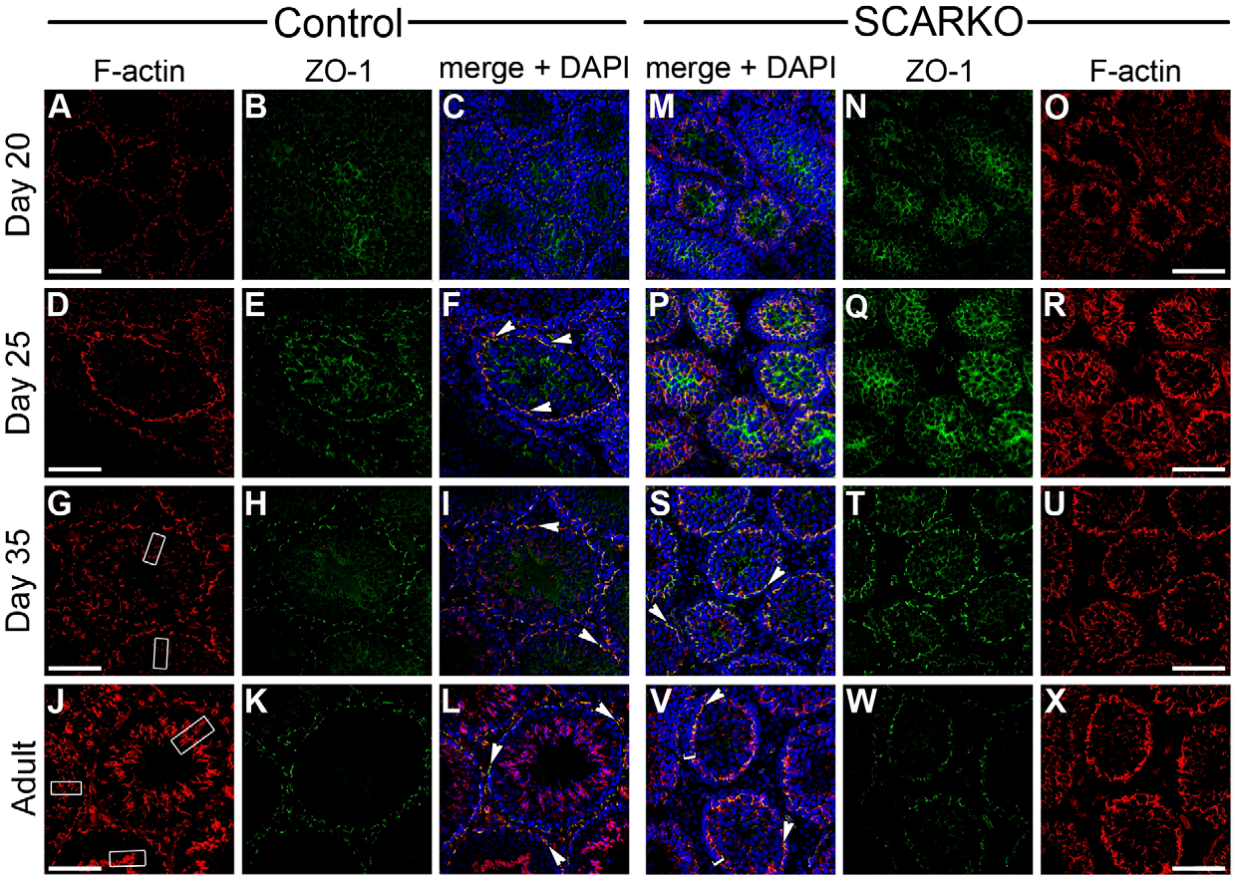
Evolution of SC barrier formation in testes of SCARKO and control mice. SC barrier formation was studied by combined staining for zonula occludens 1 (ZO-1; green) and filamentous actin (F-actin; red) on frozen sections of testes derived from SCARKO and control littermates at the indicated ages as described in the Methods section. Localization of F-actin (panel A, D, G and J) and ZO-1 (panel B, E, H and K) in testes from control mice is shown at day 20 (panel A, B), day 25 (panel D, E), day 35 (panel G, H) and at adult age (panel J, K). The corresponding merged images of F-actin, ZO-1 as well as of DAPI (blue) staining are shown in panels C (day 20), F (day 25), I (day 35) and L (adult). Localization of F-actin (panel O, R, U and X) and ZO-1 (panel N, Q, T and W) in testes from SCARKO mice is shown at day 20 (panel O, N), day 25 (panel R, Q), day 35 (panel U, T) and at adult age (panel X, W). The corresponding merged images of F-actin, ZO-1 as well as of DAPI (blue) staining are shown in panels M (day 20), P (day 25), S (day 35) and V (adult). For each genotype 3 animals were studied at each time point. Colocalization of F-actin and ZO-1 (yellow staining; white arrowheads) in wavy bands parallel with the basal lamina, consistent with SC barrier formation, may be noted in control animals from day 25 on and in SCARKO mice from day 35 on. Also note a widening of the basal compartment in adult SCARKO testes (panel V: brackets). Prominent F-actin staining can also be observed in the central region of the tubules of control mice coinciding with apical ectoplasmic specializations (panel G, J: white boxes). Scale bars = 100 µm.

The expression of proteins associated with junctional complexes was examined using double fluorescent immunohistochemistry so as to determine both whether expression of proteins could be detected but also whether they became organized into complexes at appropriate locations within the seminiferous epithelium. Combined staining for the adaptor protein ZO-1 and F-actin demonstrated that both proteins were expressed in testes from control and SCARKO animals (Figure 4). In control mice colocalization of ZO-1 and F-actin was observed, parallel with the basal lamina, reflecting the organization/maturation of complexes within the SC barrier. This colocalization (Figure 4F, I, L: white arrowheads) became particularly prominent from day 25 on. As expected, F-actin staining was observed not only at the level of the tight junctions but also at the level of the apical ectoplasmic specializations (Figure 4G, J: white boxes). In SCARKO testes, staining for ZO-1 was quite diffuse until day 35 (Figure 4N, Q). Prominent staining of F-actin could be observed, however at the base of the tubules (Figure 4O, R, U, X). From day 35 onwards, colocalized staining for ZO-1 and F-actin, suggesting barrier formation, could clearly be observed at the base of the tubules despite residual ZO-1 staining in the center (Figure 4S). Barrier formation was even more prominent in the adult testis but junctions were located further away from the periphery of the tubules than in littermate controls (Figure 4V: brackets).

Combined staining for CX43 (gap junctions) and ESPN (basal and apical ectoplasmic specializations) also revealed expression of both proteins and provided evidence of delayed organization of junctions in SCARKO testes (Figure S2). On day 20 only diffuse staining was noted in SCARKO as well as control testes (Figure S2M, N, O and A, B, C respectively). In 25-day-old controls, colocalized staining of ESPN and CX43 was noted, parallel with the basal lamina in a location appropriate for the formation of the SC barrier complex (Figure S2F: white arrowheads). ESPN staining was also noted within the adluminal compartment and was particularly prominent around the heads of the elongated spermatids from day 35 on, consistent with the formation of apical ectoplasmic specializations (Figure S2H, K). In 25- and 35-day-old SCARKO mice both ESPN (Figure S2Q, T) and CX43 (Figure S2R, U) staining appeared as a non-homogenous filiform network within the tubules with only sporadic indications of more intensive accumulations near the base of the tubules. In adult SCARKO mice many tubules showed tortuous strands of colocalized CX43 and ESPN, mostly oriented perpendicular rather than parallel to the basal lamina (Figure S2V: white arrowheads).

**Figure 5:**
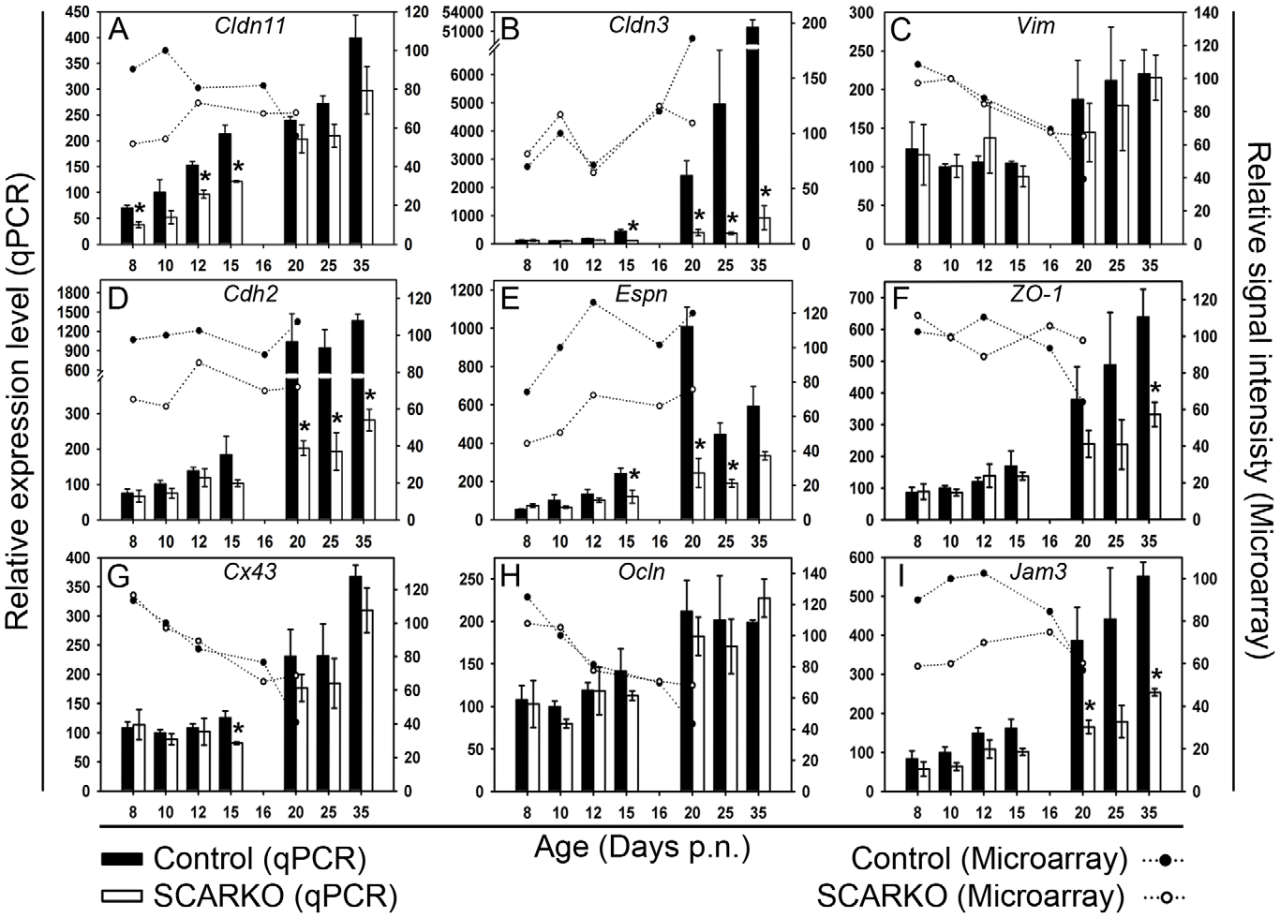
mRNA expression profiles of genes related to tight junction components and intermediate filaments. Testicular transcript levels for *Cldn11* (panel A), *Cldn3* (panel B), *Vim* (panel C), *Cdh2* (panel D), *Espn* (panel E), *ZO-1* (panel F), *Cx43* (panel G), *Ocln* (panel H) and *Jam3* (panel I) were measured by qPCR and corrected for exogenously added *luciferase* mRNA as described in the Methods section. qPCR data (bars) represent the mean ± SEM of measurements on 3 SCARKO and 3 control testes studied on day 8, 10, 12, 15, 20, 25 and 35 postnatal (p.n.). The mean transcript level of the control measured on day 10 was arbitrarily assigned a value of 100. Statistically significant differences between SCARKO and control animals are indicated by an asterisk. The original microarray data (lines) are shown for comparison. Microarray measurements were performed on a pool of mRNA derived from testes of 3 SCARKO and 3 control mice (day 8, 10, 12, 16, 20 p.n.) [19]. Values (relative signal intensities) were expressed relative to the value of the control on day 10 (arbitrarily assigned a value of 100). Note that microarray measurements reflect transcript levels in a given amount of RNA. Due to the increasing contribution of germ cells selectively in control mice and not in SCARKO mice (which develop a block in meiosis), SC transcripts become diluted in the total amount of RNA and microarrays may fail to demonstrate differences in the expression of SC genes after day 15. The correction of the qPCR data for exogenously added *luciferase* circumvents this problem by reflecting transcript levels per testis [19].

To investigate whether changes in the expression of molecules related to tight junction formation in the testis might be involved in the disturbed development of the SC barrier in SCARKO mice we also studied the transcript levels of a number of relevant genes. Figure 5 summarizes results selected from an earlier microarray study (day 8–20) [19] and data from a new, independent and more detailed qPCR time study (day 8–35). The qPCR data revealed a marked and significant decrease in the transcript level of *Cldn3* (Figure 5B) in SCARKO testes as compared to control testes from day 15 on. A tendency towards lower expression levels in SCARKO testes was also noted for *Cdh2* (Figure 5D), *Espn* (Figure 5E) and *Jam3* (Figure 5I) from day 10 on (confirming the microarray data) but significant differences were only found from day 15 on (*Espn*) or from day 20 on (*Cdh2* and *Jam3*). Similarly, a limited decrease in the transcript level of *Cldn11* (Figure 5A) was seen over the entire period studied. Given the limited number of mice studied (n = 3) significant effects were observed only at day 8, 12 and 15. In a recent study on a larger number of mice (n = 10), however, a significant decrease in the expression of *Cldn11* in SCARKO testes was confirmed over the entire period studied (day 4–50) [23]. No or only minor differences in expression level were observed for *Cx43* (Figure 5G), *Ocln* (Figure 5H) and *Vim* (Figure 5C) (again confirming the microarray data). *ZO-1* transcript levels (Figure 5F) tended to be lower from day 20 on but significant differences were noted only on day 35.

### Abnormal positioning of SC nuclei and abnormal localization of vimentin in SCARKO testes

**Figure 6:**
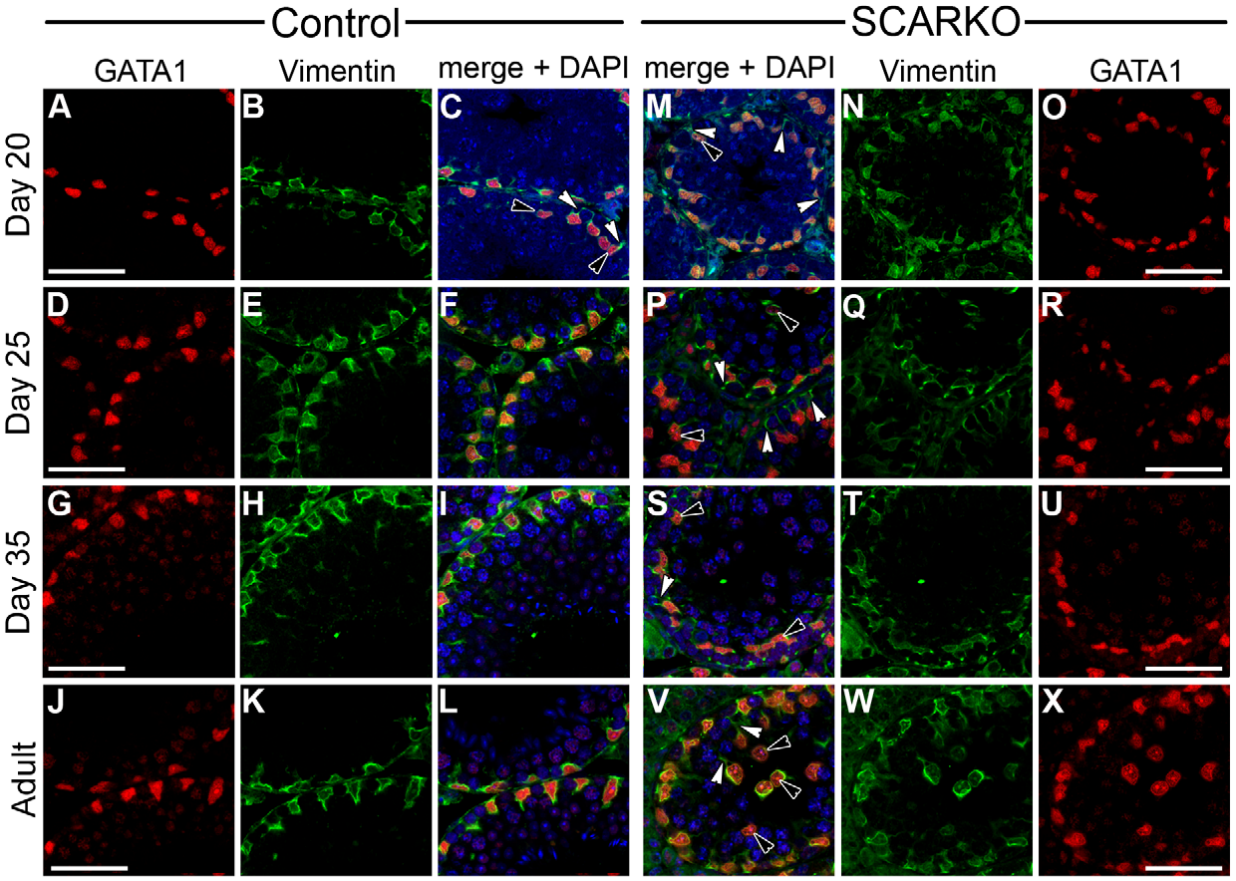
Localization of SC nuclei and associated cytoskeleton in testes of SCARKO and control mice. Testes were derived from SCARKO mice and control littermates at the indicated ages. SC nuclei and the associated cytoskeleton were studied by immunostaining for GATA binding protein 1 (GATA1; red) and the intermediate filament protein vimentin (VIM; green) respectively. Expression of GATA1 (panel A, D, G and J) and VIM (panel B, E, H and K) in testes from control mice is shown at day 20 (panel A, B), day 25 (panel D, E), day 35 (panel G, H) and at adult age (panel J, K). The corresponding merged images of GATA1, VIM as well as of DAPI (blue) staining are shown in panels C (day 20), F (day 25), I (day 35) and L (adult). Expression of GATA1 (panel O, R, U and X) and VIM (panel N, Q, T and W) in testes from SCARKO mice is shown at day 20 (panel O, N), day 25 (panel R, Q), day 35 (panel U, T) and at adult age (panel X, W). The corresponding merged images of GATA1, VIM as well as of DAPI (blue) staining are shown in panels M (day 20), P (day 25), S (day 35) and V (adult). For each genotype 3 animals were studied at each time point. In control mice SC nuclei become localized to the basal portion of the epithelium from day 25 on. Moreover, nuclei are surrounded by a prominent layer of VIM. In SCARKO mice many nuclei fail to migrate to the periphery of the tubules (black arrowheads) and anchor-like structures of VIM (white arrowheads) may be noticed connecting these nuclei to the basal lamina (as can also be seen in day 20 control mice). Scale bars = 25 µm. The data shown are representative for 2 independent time studies.

Despite the fact that important biochemical parameters of SC maturation behave similarly in SCARKO and control animals [38], the electron microscopy studies described above showed clear disturbances in nuclear maturation and localization in SCARKO testes. Combined staining for GATA1 and VIM was used to study nuclear localization and its relationship to cytoskeletal development in SCARKO and control testes (Figure 6). In 20- and 25-day-old control mice nearly all SC nuclei were found at the periphery of the tubules close to the basal lamina (Figure 6C, F). However, in SCARKO littermates of the same ages, SC nuclei were found at variable locations, often forming one- or two-layered rings positioned more centrally in the tubules (Figure 6M, P: black arrowheads). In control testes, VIM staining surrounded the peripherally located nuclei (Figure 6C, F, I, L) whereas in SCARKO testes only faint VIM staining was seen around the centrally located nuclei, but anchor-like structures with radial extensions were evident apparently connecting the SC nuclei to the basal lamina (Figure 6M, P, S, V: white arrowheads). Comparable anchor-like structures were observed in 20-day-old controls (Figure 6C: white arrowheads). Examination of testes of older control animals (day 35 or adult: Figure 6I, L) revealed further migration of SC nuclei to the periphery of the tubules and perinuclear localization of VIM with stalk-like extensions to the centre of the tubules. In SCARKO animals, progressive migration of SC nuclei to a more basal location was observed in some tubules, but in many tubules centrally located SC nuclei connected to the basal lamina by VIM anchors remained evident (Figure 6S, V).

### Novel targets of androgen action in SC related to cell adhesion and cytoskeletal dynamics

**Figure 7:**
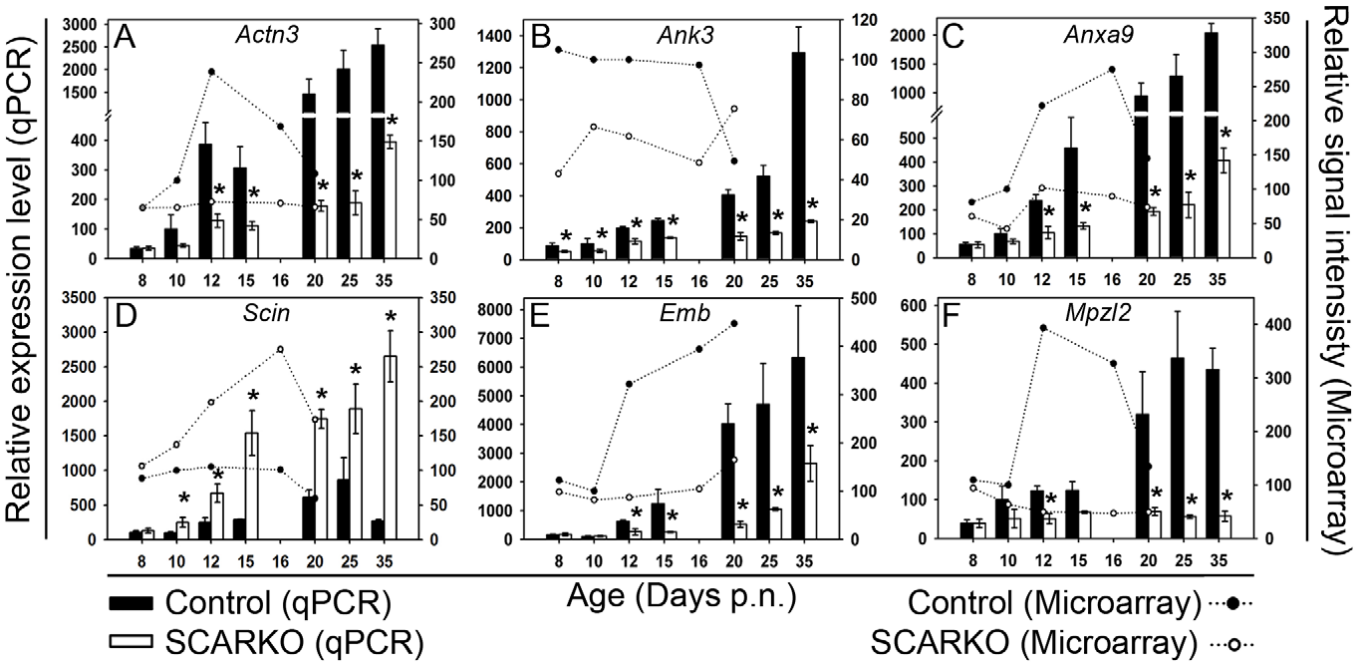
mRNA expression profiles of new candidate androgen regulated target genes. Genes related to cytoskeletal dynamics (*Actn3*: panel A; *Ank3*: panel B; *Anxa9*: panel C; *Scin*: panel D) or cell adhesion (*Emb*: panel E; *Mpzl2*: panel F) were selected based on their expression profile in a microarray time study (day 8–20) [19]. Transcript levels were measured by qPCR and corrected for exogenously added *luciferase* mRNA as described in the Methods section. qPCR data (bars) represent the mean ± SEM of measurements on 3 SCARKO and 3 control testes studied on day 8, 10, 12, 15, 20, 25 and 35 postnatal (p.n.). The mean transcript level of the control measured on day 10 was arbitrarily assigned a value of 100. Statistically significant differences between SCARKO and control animals are indicated by an asterisk. The original microarray data (lines) are shown for comparison. Microarray measurements were performed on a pool of mRNA derived from testes of 3 SCARKO and 3 control mice (day 8, 10, 12, 16, 20 p.n.). Values (relative signal intensities) were expressed relative to the value of the control on day 10 (arbitrarily assigned a value of 100).

Previous studies have focused on the potential role of molecules that are already known to play a role in tubular restructuring (including the above described cell adhesion molecules and cytoskeletal elements). In search for novel and potentially relevant targets for androgen action we screened the data from an earlier microarray study comparing transcript levels in SCARKO and control animals between day 8 and 20 [19] for genes displaying striking differential expression patterns. Particular attention was paid to genes involved in cell adhesion and cytoskeletal dynamics. The original microarray data and an independent and more detailed qPCR time study for 6 potentially relevant genes are summarized in Figure 7. Four of the studied genes (*Actn3*, *Ank3*, *Anxa9* and *Scin*) have been related to cytoskeletal remodeling, two of them (*Emb*, *Mpzl2*) encode cell adhesion molecules. Apart from *Scin* that showed significantly higher expression levels in SCARKO testes, all the other genes studied were down-regulated in SCARKO mice. The qPCR data essentially confirmed the differences in expression level suspected from the microarray data. For the 6 genes investigated, a tendency towards differential expression was evident from day 10 or from day 12 (*Emb*) onwards. Statistically significant differences were noted from day 8 on for *Ank3* (Figure 7B), from day 10 on for *Scin* (Figure 7D) and from day 12 on for *Actn3* (Figure 7A), *Anxa9* (Figure 7C), *Emb* (Figure 7E) and *Mpzl2* (Figure 7F). The microarray data suggest a decrease in transcript levels after day 15 for most of the genes studied. As previously discussed [19], this is an artifact caused by the increasing contribution of developing GC to the total amount of RNA, particularly in controls. This increase is not observed in the qPCR data since these data are corrected for exogenously added *luciferase* and accordingly reflect transcript levels per testis.

To confirm that the described genes are expressed in SC, expression levels were compared in whole testis extracts and in enzymatically prepared fractions enriched for interstitial or tubular cells (Data S1; Figure S3). Part of the latter fraction was subjected to hypotonic shock to eliminate GC and to obtain further enrichment for SC. *Rhox5*, *Sycp3* and *Cyp17a1* were included as typical representatives of genes expressed in SC, GC and interstitial (Leydig) cells respectively. It should be noted that, like *Rhox5*, the 9 selected genes displayed lower expression levels in the interstitial than in the tubular cell fraction and that hypotonic treatment of the latter fraction caused a further increase in transcript levels suggesting that SC are the main source of the studied transcripts.

## Discussion

The data summarized here show that selective ablation of the AR in SC affects SC maturation, results in delayed and incomplete formation of the SC barrier, and changes the expression pattern of several genes related to cell adhesion and cytoskeletal development in SC.

SC maturation is a complex process that may be influenced by hormones (including androgens, FSH, thyroid hormone…), local regulatory factors (including growth factors, cytokines and interactions with neighboring somatic cells and GC) and testicular localization (intrascrotal or cryptorchid) [25], [39]–[44]. Testicular androgens undoubtedly play a crucial role but the nature of their effects and the underlying molecular and cellular mechanisms remain only partially understood. Some 30 years ago already it was noted that interference with androgen production during the first 32 days of life precluded the expected maturational changes in the nucleus, nucleolus and cytoplasm of rat SC without affecting the development of SC tight junctions [45]. The SCARKO model offers a unique opportunity to explore whether these effects depend on cell-autonomous activation of the AR in SC.

Surprisingly, initial observations in the SCARKO model indicated that several important parameters reflecting SC maturation develop normally in the absence of AR expression in SC and even in the general absence of AR expression (ARKO mice with ubiquitous ablation of the AR) [38]. As in WT animals, SC in SCARKO and ARKO mice show a rapid decline in anti-Müllerian hormone (AMH) expression during the first weeks of life and an early pubertal rise in the expression of cyclin-dependent kinase inhibitor 1B (CDKN1B or p27^kip1^; marking the end of SC proliferation), GATA1 (an important transcription factor in SC) and clusterin (CLU or SGP2; one of the main SC secreted proteins). Nonetheless, SCARKO SC are obviously unable to support completion of meiosis and spermatid formation and, at the onset of meiosis (day 10), their gene expression pattern differs markedly from that observed in testes and SC from normal controls [19]. The data presented here provide novel information and further support the contention that activation of the AR in SC is mandatory to allow the changes in tubular architecture and junction dynamics that accompany normal tubular development and that are needed to allow initiation of spermatogenesis.

Evaluation of SC barrier formation by 3 different techniques (hypertonic perfusion, examination of lanthanum permeation by electron microscopy and immunohistochemical analysis) indicates that barrier formation is a progressive process in which not all aspects may be completed at the same time. In control mice, hypertonic perfusion experiments and lanthanum permeability studies suggest initial barrier formation from day 15 onwards, whereas immunohistochemical studies indicate that complete organization of tight junction complexes may take at least 10 more days. Although differences in sensitivity of the various techniques cannot be excluded, these findings are reminiscent of earlier observations in the rat showing that hypertonic perfusion or lanthanum penetration experiments point to the formation of a functional barrier between day 16 and 19 whereas a more quantitative evaluation based on the penetration of labeled Cr-EDTA or albumin indicates that it may take until day 44 before the tightness of the adult barrier is achieved [32], [46]. Our experiments in the SCARKO mouse model show unequivocally that an active AR in SC is mandatory for timely and complete barrier formation. These findings confirm and extend earlier data showing a defective barrier in *Tfm* mice or Ar^flox(ex1-neo)/Y^; AMH-Cre mice, two models in which AR ablation is not limited to SC [28], [30]. Hypertonic perfusion studies indicate that many tubules in the SCARKO still form a barrier that protects adluminally located cells from shrinkage. The formation of this barrier, however, is clearly delayed (by 5–10 days). Furthermore, despite indications of the presence of a barrier some tubules display regional shrinkage of adluminally located GC suggesting that at least at some places the barrier must be leaky or incomplete. Electron microscopy and lanthanum permeation studies confirm the presence of functional tight junctions in SCARKO testes. Interestingly, however, these junctions are not found between the most peripherally located SC that apparently show signs of immaturity, but between SC that are located more centrally in the tubules and that display a higher degree of maturation. Here too, studies in 35-day-old and adult SCARKO testes indicate that, despite the formation of tight junctions, lanthanum may be seen in the adluminal compartment of some tubules. Immunohistochemical evaluation of the development of the SC barrier in control and SCARKO mice further confirms the delayed and incomplete barrier formation in the SCARKO testes. In control mice ‘wavy bands’ of colocalized CX43 and ESPN as well as ZO-1 and F-actin were localized parallel with and close to the basal lamina from day 25 onwards. In SCARKO mice, colocalization of ZO-1 and F-actin at the base of the tubules only became evident from day 35 onwards and colocalization of CX43 and ESPN was observed only in the adult testis. Moreover, intense immunostaining for these proteins was located further from the periphery of the tubule and perpendicular to the basal lamina rather than parallel to it in SCARKO mice on and after day 35. A similar formation of lanthanum-impermeable junctions perpendicular to the basal lamina has been described in rats after prenatal treatment with busulphan [47].

At present we can only speculate on the mechanisms by which androgens may affect SC barrier formation. Earlier studies suggested that androgens may be essential for the expression of *Cldn3*, a junction protein that associates specifically with newly formed tight junctions and that, according to recent data, might act as a sealing component that delineates the transiently existing translocation compartment allowing transfer of leptotene spermatocytes through the SC barrier [30], [48]. Our data confirm markedly decreased *Cldn3* transcript levels in SCARKO testes from day 15 onwards but also show that differences in transcript levels of other tight junction elements such as *Cldn11* and *Jam3* are already evident at a much earlier time points (days 4 and 10 respectively) [23]. *Cldn11* is known to be essential for tight junction formation and male fertility [49] and the dependence of its expression on androgens has been documented also in other studies [22], [50], [51]. However it is well established that the mature basal SC barrier consists of multiple junctional complexes with multiple protein constituents (see reviews by Cheng and co-workers [26], [27], [52]) and from our gene expression data it is evident that, apart from the claudins, the expression of *Cdh2* and *Espn* (and to a lesser extent *ZO-1*) are all impaired in SCARKO mice whereas no consistent differences in transcript levels were observed for *Cx43* and *Ocln*. In addition, it should be noted that the SC barrier is a very dynamic structure and that apart from transcript levels also protein levels and posttranslational modifications affecting localization and interaction between the various components may be important for correct function. The immunohistochemical data shown in this study clearly support the contention that androgens not only affect the level of expression of tight junction related molecules but also their distribution/localization. Recent data have stressed the essential role of ether-lipids in barrier dynamics and correct positioning of tight junction proteins [48]. Given the well-known effects of androgens on lipogenesis [53], it would be worthwhile to study whether androgens affect ether lipid metabolism in SC. Furthermore, and along the same lines, it has been demonstrated that androgens may alter barrier dynamics by affecting the kinetics of endocytosis and recycling of barrier-related proteins [52].

Our data confirm earlier observations indicating a parallelism between barrier formation and initiation of meiosis [32]. Given the increasing evidence for bilateral interactions between GC and SC in the dynamics of barrier function [27], [54], this raises the question whether androgens might affect barrier development indirectly by promoting meiotic progression. No unambiguous answer can be provided to this important issue at the present time. Few studies have addressed barrier formation in mice with a primary defect in GC affecting meiotic progression. Normal barrier formation -as judged from perfusion with a hypertonic fixative- was observed in mice with the Weaver mutation that causes a variable disturbance in GC development with some tubules containing only spermatogonia and others showing only absence of elongated spermatids [55]. Also in W/W^v^ mice [56] in which no spermatogenic cells are present in the seminiferous epithelium it has been demonstrated that SC junction formation develops normally [57] and that SC cytology and cytoskeleton are only minimally affected [58]. Lumen formation, suggesting polarized secretion by SC, has been observed in *Dazl^−/−^* mice that show a spermatogenic arrest at the leptotene stage of meiotic prophase I [59]. Similarly, inter-SC tight junction formation has been shown to remain intact in rats made GC depleted by a vitamin A deficient diet [60]. Conversely, increased permeability of the SC barrier and impaired tissue remodeling have recently been described in mice in which terminal differentiation of SC fails due to a SC-selective inactivation of the retinoblastoma protein [61]. Taken together these data suggest that at least part of the defect in barrier formation in SCARKO mice may directly be caused by defective SC function.

One of the other defects observed in SC lacking AR (apart from their inability to support timely and complete barrier formation and progression through meiosis) is the failure of the nucleus to adopt its typical position close to the basal lamina. This defect was also noticed by other investigators [22]. In this paper analysis of SC maturation in immature SCARKO mice using electron microscopy points to a failure of nuclear maturation. Signs of immaturity, such as irregular nuclear shape and condensed chromatin deposits on the nuclear membrane, have previously been described in SCARKO-*Jsd* mice [62]. Unexpectedly, in pubertal SCARKO mice more basally located nuclei tend to show more signs of immaturity than centrally located nuclei. The mechanisms underlying this maturational defect remain unclear. Given the alleged role of the cytoskeleton in the polarization of SC and in the positioning of its major organelles [63], and given the indications that the AR may affect cytoskeletal dynamics in SC [15], we investigated the expression and localization of vimentin in developing SC. Over the entire period studied (day 8–35) we did not observe decreased transcript levels for vimentin as claimed by other investigators [22]. Nonetheless, clear differences in the localization of vimentin staining were seen in SC of SCARKO and control mice. In control animals the vimentin cytoskeleton surrounded the SC nuclei with extensions directed towards the center of the tubules. In the SCARKO testis, and particularly in cells with centrally located nuclei, vimentin was mainly found in anchor-like structures connecting the lower pole of the nucleus to the most peripheral part of the tubule close to the basal lamina. Whether this change is causally related to the absence of androgen action or whether it is just a reflection of the lack of normal cell polarization remains to be investigated.

A search for new potentially relevant androgen target genes related to cell adhesion and cytoskeletal dynamics that might be differentially expressed in SCARKO and control animals during early puberty, revealed at least 6 candidates. ACTN3 (actinin-α3) is a cytoskeletal actin-binding protein and a member of the spectrin superfamily. It is found in anchoring junctions and, besides binding to actin filaments, it associates with cytoskeletal elements, signaling molecules and with the cytoplasmic domains of transmembrane proteins and ion channels [26], [64]. ANK3 (ankyrin 3) is a member of the ankyrin protein family and links integral membrane proteins to the spectrin-based cytoskeleton. It is thought to play a role in the polarized distribution of proteins to specific subcellular sites [65]. ANXA9 (annexin A9) belongs to the annexins, a family of evolutionary conserved proteins characterized by their ability to interact with membrane phospholipids in a Ca^++^-dependent way. These proteins have been implicated in membrane organization, membrane-cytoskeleton contacts and vesicular transport. A unique feature of ANXA9 is that its Ca^++^-binding sites are dysfunctional and accordingly its function is unknown [66]. Interestingly, its promoter binds GATA1 [67], an important transcription factor in SC, the expression of which we monitored in our samples. SCIN (scinderin) is an actin-severing protein reported typically in tissues demonstrating a high secretory activity. In bovine SC it was shown to accumulate within the cytoplasm near the base of the cells in a stage-specific manner suggesting a potential role in the regulation of tight junction permeability [68]. EMB (embigin) is a cell adhesion molecule belonging to the immunoglobulin superfamily and involved in cell-extracellular matrix interactions during development [69]. Increased expression has been correlated with the appearance of highly organized luminal and ductal structures [70]. Its congener basigin (ablation of which results in male infertility) also seems to be involved in the translocation of monocarboxylate transporters to the plasma membrane [71], [72]. MPZL2 (myelin protein zero-like 2) is also a member of the immunoglobulin superfamily expressed in various epithelia. It has been shown to mediate cell adhesion through homophilic interaction and some data suggest association with the cytoskeleton [73]. Interestingly, recent data point to a role in the control of the permeability of the blood-cerebrospinal fluid barrier [74]. For all of these genes, we present evidence that SC are their main or only site of cellular localization, indicating that they may contribute to the observed effects of the SC AR on cell maturation, cell interactions and tubular restructuring.

In conclusion, targeted ablation of AR from SC does not completely prevent the formation of an anatomical and functional barrier defining basal and adluminal compartments within the seminiferous epithelium. However, barrier formation is delayed and defects are observed in many tubules. The defective barrier formation is accompanied by disturbances in the nuclear maturation process and in SC polarization resulting in aberrant positioning of cytoskeletal elements such as vimentin. The observed developmental defects in SCARKO SC are accompanied by disturbances in the expression of well known molecules involved in cell adhesion and cytoskeleton building but marked differences were also observed in a number of previously unstudied molecules that may be related to these functions. These findings support the contention that absence of the AR in SC has major implications for the pubertal and postpubertal events of tubular restructuring that are essential to allow normal initiation and progression of spermatogenesis.

## Supporting Information

Data S1 (0.04 MB DOC)

Figure S1 Quantitative evaluation of the formation of a functional SC barrier in SCARKO and control testes. Testes derived from SCARKO and control mice at the indicated ages (day 25, day 35 and adult; at least 3 animals at each time point) were evaluated for the presence of a functional SC barrier after perfusion with a hypertonic solution. Testes were embedded, sectioned and stained as described in Materials and Methods. SC barrier formation was evaluated by light microscopy for the indicated numbers (n) of tubular sections. Barrier formation was scored as 'fully functional' when hypertonicity-induced shrinkage was limited to cells in the basal compartment (panel A), as 'intermediate' when shrinkage was not limited to the basal compartment but was also observed in other peripherally located cells (panel B) and as 'defective' when shrinkage was also seen in centrally located cells (panel C). Results are summarized in panel D. Barrier formation is delayed in SCARKO tubules but the number of tubules showing an intermediate/defective barrier decreases as a function of age. The scale bar in panel A, B and C represents 50 μm.
(1.31 MB TIF)

Figure S2 SC barrier formation in SCARKO and control testes studied by combined staining for connexin 43 and espin. SC barrier formation was studied by combined staining for the gap junction protein connexin 43 (CX43; red) and for espin (ESPN; green) a marker of basal and apical ectoplasmic specializations, as explained in Materials and Methods. Localization of CX43 (panel A, D, G and J) and ESPN (panel B, E, H and K) in testes from control mice is shown at day 20 (panel A, B), day 25 (panel D, E), day 35 (panel G, H) and at adult age (panel J, K). The corresponding merged images of CX43, ESPN as well as of DAPI (blue) staining are shown in panels C (day 20), F (day 25), I (day 35) and L (adult). Localization of CX43 (panel O, R, U and X) and ESPN (panel N, Q, T and W) in testes from SCARKO mice is shown at day 20 (panel O, N), day 25 (panel R, Q), day 35 (panel U, T) and at adult age (panel X, W). The corresponding merged images of CX43, ESPN as well as of DAPI (blue) staining are shown in panels M (day 20), P (day 25), S (day 35) and V (adult). For each genotype 3 animals were studied at each time point. In controls ESPN and CX43 are colocalized (yellow staining; white arrowheads), parallel with the basal lamina, in a location appropriate for the SC barrier, from day 25 on (panel F, I and L). In the adult SCARKO (panel V), colocalized ESPN and CX43 (white arrowheads) may be noted as tortuous strands mostly oriented perpendicular rather than parallel to the basal lamina. Scale bars  =  50 μm.
(2.84 MB TIF)

Figure S3 Testicular localization of transcripts of genes related to the cytoskeleton or cell adhesion. Testes from 20-day-old mice were separated in an interstitial fraction and a tubular fraction by enzymatic digestion as described in Data S1. Part of the tubular fraction was subjected to hypotonic treatment to destroy germinal cells and enrich SC. Transcript levels were measured by qPCR in RNA extracts from whole testes, interstitial fraction, tubular fraction and tubular fraction enriched for SC (indicated as hypotonic shock). All measurements were corrected for Rn18S. The transcript level measured in whole testis extract was arbitrarily assigned a value of 100 and relative expression levels were calculated for the other fractions. The depicted genes include a marker for SC (Rhox5: panel A), GC (Sycp3: panel B) and Leydig cells (Cyp17a1: panel C), cytoskeletal genes (Actn3: panel G; Ank3: panel H; Anxa9: panel I and Scin: panel J) and genes encoding cell adhesion molecules (Emb: panel K; Mpzl2: panel L; Cldn11: panel D; Cldn3: panel E and Jam3: panel F). One representative experiment out of three independent experiments is shown.
(0.58 MB TIF)

Table S1 Change in testis weight after efferent duct ligation in SCARKO and control mice. Testis weight was measured 24 h after efferent duct ligation (EDL) in SCARKO and control mice of the indicated ages. Efferent duct ligation was performed as described [4]. In control animals a weight gain (approximately 19 %) was consistently observed in the ligated testis as compared to the contralateral sham operated testis. In SCARKO testes the weight of the ligated testis tended to be lower than that of the unligated testis. Statistical analysis (paired t-test) on the pooled data of 74- and 380-day-old mice revealed a significant (p ≤ 0.05) difference in ligated and unligated testis weight for the control animals (125.4 ± 7.2 mg (mean ± SEM) and 105.3 ± 4.2 mg respectively). The difference in weight between the ligated and unligated SCARKO testes (32.0 ± 3.0 mg and 33.3 ± 2.6 mg) was not statistically significant.
(0.05 MB DOC)
