# Supplementary material for: Selective Ablation of the Androgen Receptor in Mouse Sertoli Cells Affects Sertoli Cell Maturation, Barrier Formation and Cytoskeletal Development
Source: PLoS One. 2010 Nov 30;5(11):e14168. doi: 10.1371/journal.pone.0014168 (PMC2994754; doi:10.1371/journal.pone.0014168)
Supplement: Data S1 — (0.04 MB DOC) [file pone.0014168.s001.doc]

**Data S1**

Enzymatic separation of interstitial and tubular fractions. Enrichment for Sertoli cells by hypotonic shock

Interstitial and tubular fractions were prepared by enzymatic digestions essentially as described previously [1]. Hypotonic treatment of the tubular fractions was basically performed as described by Galdieri *et al.* [2].

Briefly, a pool of 40 testes, derived from 20-day-old C57BL/6 mice (Janvier), were decapsulated. Three testes were randomly removed from this pool to serve as “whole testis fraction”. The remaining testes were placed in DPBS without MgCl_2_ (Invitrogen), supplemented with Penicillin-Streptomycin (100 U/ml and 100 µg/ml respectively; Invitrogen) and D-glucose (1.5 mg/ml; Merk) -further referred to as PBS-A- and were then put in a shaking water bath (120 oscillations/min) at 32°C for 10 min. Next, tubules were left to sediment for 10 min and supernatant was collected as part of the “interstitial fraction”. Subsequently, the sedimented tubules were digested for 10 min with Trypsin (1 mg/ml; SIGMA) and DNase I (10 µg/ml; Roche) in a shaking water bath (120 oscillations/min, 32°C). The action of trypsin was stopped by the addition of Soybean Trypsin Inhibitor (1.3 mg/ml; SIGMA). After sedimentation of the tubular fragments, the supernatant was collected as the remainder of the “interstitial fraction”. The sedimented tubular fragments were again treated with Soybean Trypsin Inhibitor (1 mg/ml) and were subsequently washed 3 times with PBS-A for 10 min. Then tubules were treated for 5 min with collagenase (1 mg/ml; Roche), hyaluronidase (1 mg/ml; SIGMA), and DNase I (10 µg/ml) in a shaking water bath (120 oscillations/min; 32°C) to remove peritubular myoid cells. The tubular fraction was left to sediment for 10 min, supernatant was removed, the tubular fraction was washed 4 times with PBS-A and treated once more with hyaluronidase (1 mg/ml) for 15 min in a shaking water bath (120 oscillations/min; 32°C). Again, the tubular fraction was allowed to sediment for 10 min and supernatant was removed. The sediment was washed 3 times with PBS-A. Thereafter 17 ml RPMI-A (containing 25 mM HEPES and 5 mM L-glutamine added to RPMI; Invitrogen) was added and cells were dispersed with a needle (18 G). Two ml of this suspension was collected as “tubular fraction”. The remaining suspension was centrifuged (199 x *g*; 10 min), the supernatant was removed and the pellet was exposed to hypotonic treatment by adding 5 ml 20 mM Tris-HCl for 1 min [2]. The hypotonic treatment was stopped by adding 45 ml RPMI-A and the remaining cells (mainly Sertoli cells) were collected by centrifugation at 40 x *g* for 4 min [3]. Pelleted cells were washed one more time with PBS-A and collected as the “SC enriched fraction”.

The collected fractions were centrifuged (199 x *g*; 10 min) -except for the whole testis fraction-, resuspended in RLT buffer from the Qiagen RNeasy® Mini kit, snap-frozen in liquid nitrogen and stored at -80°C for RNA preparation. RNA was isolated using the Qiagen RNeasy® Mini kit with an on column DNase I treatment as described in the Materials and Methods section.

References

1. Hoeben E, Swinnen JV, Heyns W, Verhoeven G (1999) Heregulins or neu differentiation factors and the interactions between peritubular myoid cells and Sertoli cells. Endocrinology 140: 2216-2223.

2. Galdieri M, Ziparo E, Palombi F, Russo MA, Stefanini M (1981) Pure Sertoli-cell cultures - a new model for the study of somatic-germ cell-interactions. J Androl 2: 249-254.

3. Anway MD, Folmer J, Wright WW, Zirkin BR (2003) Isolation of Sertoli cells from adult rat testes: An approach to ex vivo studies of Sertoli cell function. Biol Reprod 68: 996-1002.

4. Russell LD, Bartke A, Goh JC (1989) Postnatal-development of the Sertoli-cell barrier, tubular lumen, and cytoskeleton of Sertoli and myoid cells in the rat, and their relationship to tubular fluid secretion and flow. Am J Anat 184: 179-189.
