# Supplementary figures and images for: Selective Ablation of the Androgen Receptor in Mouse Sertoli Cells Affects Sertoli Cell Maturation, Barrier Formation and Cytoskeletal Development
Source: PLoS One. 2010 Nov 30;5(11):e14168. doi: 10.1371/journal.pone.0014168 (PMC2994754; doi:10.1371/journal.pone.0014168)

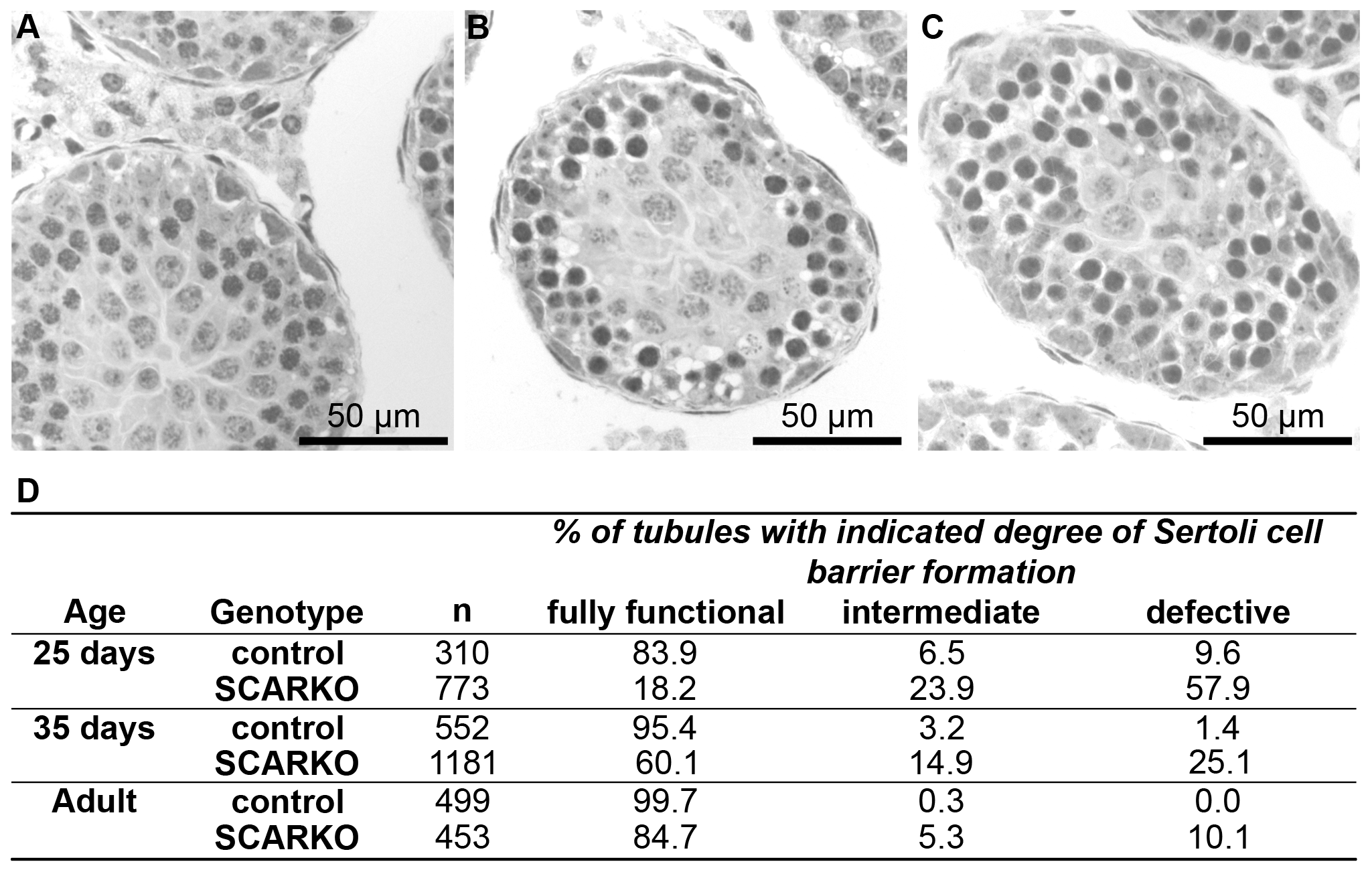

Supplement: Figure S1 — Quantitative evaluation of the formation of a functional SC barrier in SCARKO and control testes. Testes derived from SCARKO and control mice at the indicated ages (day 25, day 35 and adult; at least 3 animals at each time point) were evaluated for the presence of a functional SC barrier after perfusion with a hypertonic solution. Testes were embedded, sectioned and stained as described in Materials and Methods. SC barrier formation was evaluated by light microscopy for the indicated numbers (n) of tubular sections. Barrier formation was scored as 'fully functional' when hypertonicity-induced shrinkage was limited to cells in the basal compartment (panel A), as 'intermediate' when shrinkage was not limited to the basal compartment but was also observed in other peripherally located cells (panel B) and as 'defective' when shrinkage was also seen in centrally located cells (panel C). Results are summarized in panel D. Barrier formation is delayed in SCARKO tubules but the number of tubules showing an intermediate/defective barrier decreases as a function of age. The scale bar in panel A, B and C represents 50 μm. (1.31 MB TIF) [file pone.0014168.s002.tif]

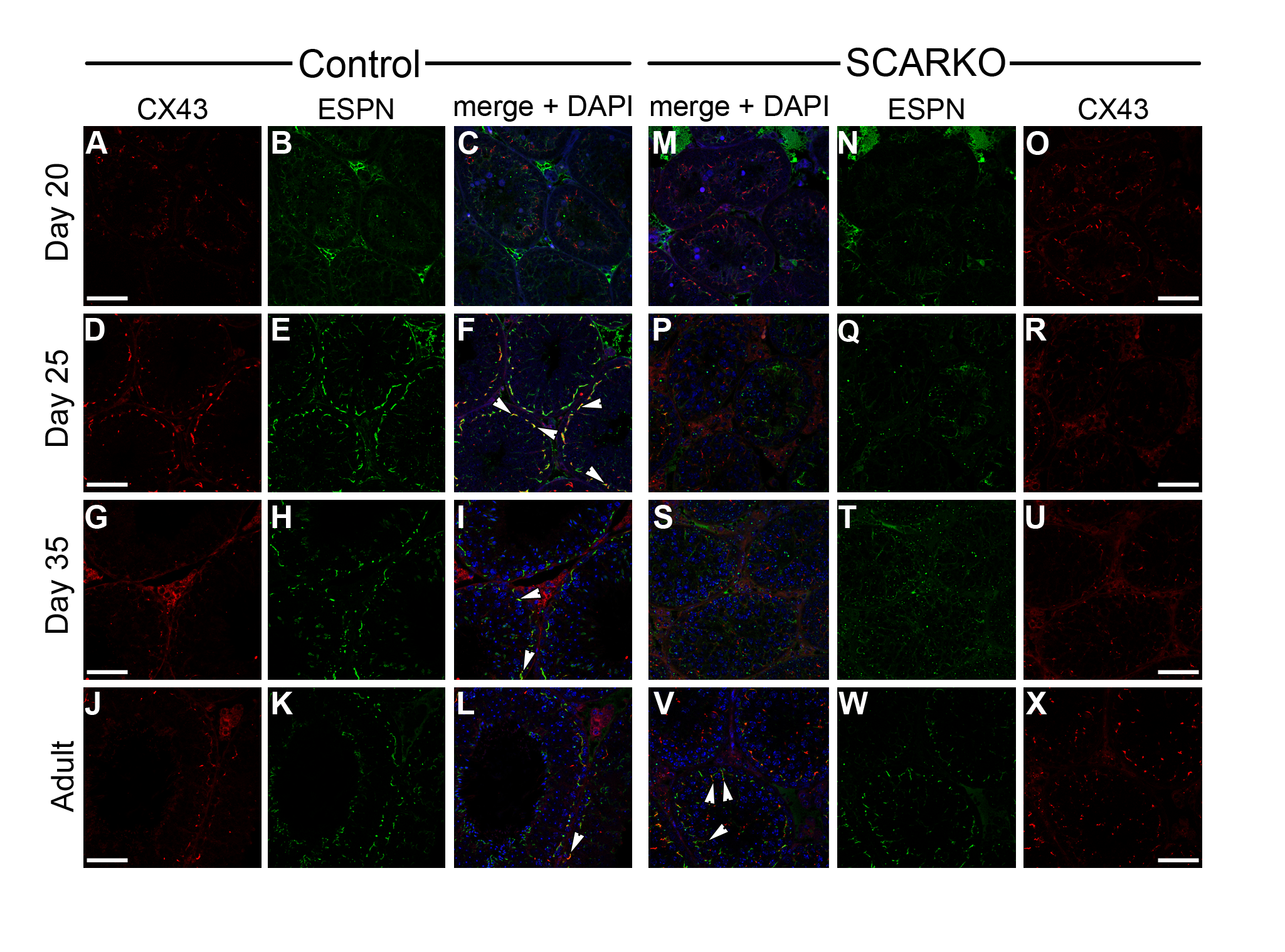

Supplement: Figure S2 — SC barrier formation in SCARKO and control testes studied by combined staining for connexin 43 and espin. SC barrier formation was studied by combined staining for the gap junction protein connexin 43 (CX43; red) and for espin (ESPN; green) a marker of basal and apical ectoplasmic specializations, as explained in Materials and Methods. Localization of CX43 (panel A, D, G and J) and ESPN (panel B, E, H and K) in testes from control mice is shown at day 20 (panel A, B), day 25 (panel D, E), day 35 (panel G, H) and at adult age (panel J, K). The corresponding merged images of CX43, ESPN as well as of DAPI (blue) staining are shown in panels C (day 20), F (day 25), I (day 35) and L (adult). Localization of CX43 (panel O, R, U and X) and ESPN (panel N, Q, T and W) in testes from SCARKO mice is shown at day 20 (panel O, N), day 25 (panel R, Q), day 35 (panel U, T) and at adult age (panel X, W). The corresponding merged images of CX43, ESPN as well as of DAPI (blue) staining are shown in panels M (day 20), P (day 25), S (day 35) and V (adult). For each genotype 3 animals were studied at each time point. In controls ESPN and CX43 are colocalized (yellow staining; white arrowheads), parallel with the basal lamina, in a location appropriate for the SC barrier, from day 25 on (panel F, I and L). In the adult SCARKO (panel V), colocalized ESPN and CX43 (white arrowheads) may be noted as tortuous strands mostly oriented perpendicular rather than parallel to the basal lamina. Scale bars = 50 μm. (2.84 MB TIF) [file pone.0014168.s003.tif]

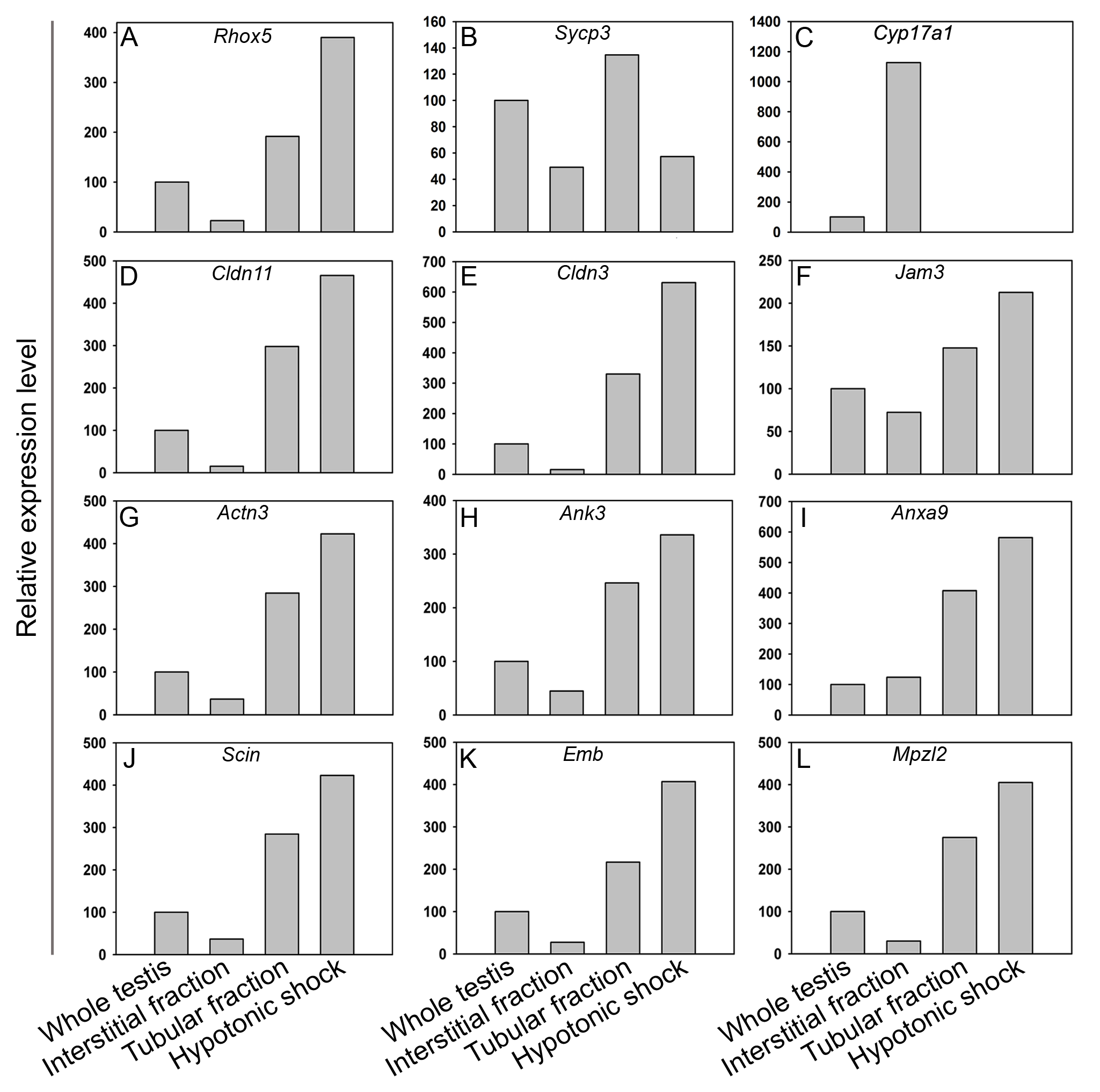

Supplement: Figure S3 — Testicular localization of transcripts of genes related to the cytoskeleton or cell adhesion. Testes from 20-day-old mice were separated in an interstitial fraction and a tubular fraction by enzymatic digestion as described in Data S1. Part of the tubular fraction was subjected to hypotonic treatment to destroy germinal cells and enrich SC. Transcript levels were measured by qPCR in RNA extracts from whole testes, interstitial fraction, tubular fraction and tubular fraction enriched for SC (indicated as hypotonic shock). All measurements were corrected for Rn18S. The transcript level measured in whole testis extract was arbitrarily assigned a value of 100 and relative expression levels were calculated for the other fractions. The depicted genes include a marker for SC (Rhox5: panel A), GC (Sycp3: panel B) and Leydig cells (Cyp17a1: panel C), cytoskeletal genes (Actn3: panel G; Ank3: panel H; Anxa9: panel I and Scin: panel J) and genes encoding cell adhesion molecules (Emb: panel K; Mpzl2: panel L; Cldn11: panel D; Cldn3: panel E and Jam3: panel F). One representative experiment out of three independent experiments is shown. (0.58 MB TIF) [file pone.0014168.s004.tif]
