# Supplementary material for: Selective Ablation of the Androgen Receptor in Mouse Sertoli Cells Affects Sertoli Cell Maturation, Barrier Formation and Cytoskeletal Development
Source: PLoS One. 2010 Nov 30;5(11):e14168. doi: 10.1371/journal.pone.0014168 (PMC2994754; doi:10.1371/journal.pone.0014168)
Supplement: Table S1 — Change in testis weight after efferent duct ligation in SCARKO and control mice. Testis weight was measured 24 h after efferent duct ligation (EDL) in SCARKO and control mice of the indicated ages. Efferent duct ligation was performed as described [4]. In control animals a weight gain (approximately 19 %) was consistently observed in the ligated testis as compared to the contralateral sham operated testis. In SCARKO testes the weight of the ligated testis tended to be lower than that of the unligated testis. Statistical analysis (paired t-test) on the pooled data of 74- and 380-day-old mice revealed a significant (p ≤ 0.05) difference in ligated and unligated testis weight for the control animals (125.4 ± 7.2 mg (mean ± SEM) and 105.3 ± 4.2 mg respectively). The difference in weight between the ligated and unligated SCARKO testes (32.0 ± 3.0 mg and 33.3 ± 2.6 mg) was not statistically significant. (0.05 MB DOC) [file pone.0014168.s005.doc]

**Table S**1. Change in testis weight after efferent duct ligation in SCARKO and control mice.

| **Age (days)** | **Genotype** | **EDL**  **weight** | **testis (mg)** | **unligated weight** | **testis (mg)** | **weight gain**  **testis** | **EDL**  **(mg)** | **% gain** |  |
| --- | --- | --- | --- | --- | --- | --- | --- | --- | --- |
| 380 | control | 136.1 |  | 113.0 |  | 23.1 |  | 20.4 |  |
|  |  | 148.7 |  | 115.3 |  | 33.4 |  | 29.0 |  |
|  |  | 117.2 |  | 106.9 |  | 10.3 |  | 9.6 |  |
|  |  | 112.8 |  | 97.1 |  | 15.7 |  | 16.2 |  |
|  |  | **mean = 128.7** | **± 8.4** | **mean = 108.1** | **± 4.1** | **mean = 20.6** | **± 5.0** | **mean = 18.8** | **± 4.1** |
| 380 | SCARKO | 30.5 |  | 32.6 |  | -2.1 |  | -6.4 |  |
|  |  | 34.1 |  | 36.8 |  | -2.7 |  | -7.3 |  |
|  |  | **mean = 32.3** | **± 1.8** | **mean = 34.7** | **± 2.1** | **mean = -2.4** | **± 0.3** | **mean = -6.9** | **± 0.4** |
| 74 | control | 112.4 |  | 94.4 |  | 18.0 |  | 19.1 |  |
|  |  | **mean = 112.4** | **± 0.0** | **mean = 94.4** | **± 0.0** | **mean = 18.0** | **± 0.0** | **mean = 19.1** | **± 0.0** |
| 74 | SCARKO | 23.2 |  | 26.5 |  | -3.3 |  | -12.5 |  |
|  |  | 26.7 |  | 27.5 |  | -0.8 |  | -2.9 |  |
|  |  | 26.0 |  | 27.7 |  | -1.7 |  | -6.1 |  |
|  |  | 26.3 |  | 28.7 |  | -2.4 |  | -8.4 |  |
|  |  | **mean = 25.6** | **± 0.8** | **mean = 27.6** | **± 0.5** | **mean = -2.1** | **± 0.5** | **mean = -7.5** | **± 2.0** |

Testis weight was measured 24 h after efferent duct ligation (EDL) in SCARKO and control mice of the indicated ages. Efferent duct ligation was performed as described [4]. In control animals a weight gain (approximately 19 %) was consistently observed in the ligated testis as compared to the contralateral sham operated testis. In SCARKO testes the weight of the ligated testis tended to be lower than that of the unligated testis. Statistical analysis (paired t-test) on the pooled data of 74- and 380-day-old mice revealed a significant (*p* ≤ 0.05) difference in ligated and unligated testis weight for the control animals (125.4 ± 7.2 mg (mean ± SEM) and 105.3 ± 4.2 mg respectively). The difference in weight between the ligated and unligated SCARKO testes (32.0 ± 3.0 mg and 33.3 ± 2.6 mg) was not statistically significant.
